# Mapping canopy nitrogen‐scapes to assess foraging habitat for a vulnerable arboreal folivore in mixed‐species *Eucalyptus* forests

**DOI:** 10.1002/ece3.8428

**Published:** 2021-12-16

**Authors:** Benjamin Wagner, Patrick J. Baker, Ben D. Moore, Craig R. Nitschke

**Affiliations:** ^1^ School of Ecosystem and Forest Sciences The University of Melbourne Richmond, Victoria Australia; ^2^ Hawkesbury Institute for the Environment The Western Sydney University Penrith, NSW Australia

**Keywords:** *Eucalyptus*, folivores, greater glider, habitat mapping, herbivory, *Petauroides volans*, plant–animal interactions, remote‐sensing spectroscopy, UAV

## Abstract

Herbivore foraging decisions are closely related to plant nutritional quality. For arboreal folivores with specialized diets, such as the vulnerable greater glider (*Petauroides volans*), the abundance of suitable forage trees can influence habitat suitability and species occurrence. The ability to model and map foliar nitrogen would therefore enhance our understanding of folivore habitat use at finer scales. We tested whether high‐resolution multispectral imagery, collected by a lightweight and low‐cost commercial unoccupied aerial vehicle (UAV), could be used to predict total and digestible foliar nitrogen (N and digN) at the tree canopy level and forest stand‐scale from leaf‐scale chemistry measurements across a gradient of mixed‐species *Eucalyptus* forests in southeastern Australia. We surveyed temperate *Eucalyptus* forests across an elevational and topographic gradient from sea level to high elevation (50–1200 m a.s.l.) for forest structure, leaf chemistry, and greater glider occurrence. Using measures of multispectral leaf reflectance and spectral indices, we estimated N and digN and mapped N and favorable feeding habitat using machine learning algorithms. Our surveys covered 17 *Eucalyptus* species ranging in foliar N from 0.63% to 1.92% dry matter (DM) and digN from 0.45% to 1.73% DM. Both multispectral leaf reflectance and spectral indices were strong predictors for N and digN in model cross‐validation. At the tree level, 79% of variability between observed and predicted measures of nitrogen was explained. A spatial supervised classification model correctly identified 80% of canopy pixels associated with high N concentrations (≥1% DM). We developed a successful method for estimating foliar nitrogen of a range of temperate *Eucalyptus* species using UAV multispectral imagery at the tree canopy level and stand scale. The ability to spatially quantify feeding habitat using UAV imagery allows remote assessments of greater glider habitat at a scale relevant to support ground surveys, management, and conservation for the vulnerable greater glider across southeastern Australia.

## Graphical abstract

For arboreal folivores such as the greater glider, which is threatened by habitat loss and climate change, the abundance of suitable forage trees can influence habitat suitability and species occurrence. We developed a method to quantify spatial variability in foliar nitrogen, a key metric in forage suitability using UAV multispectral imagery. Our approach allowed us to scale leaf‐level measures of foliar nitrogen to the stand level. This method can provide critical habitat assessments to support management and conservation of this vulnerable species across southeastern Australia.

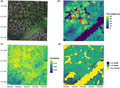

## 1 INTRODUCTION

The ability to determine and monitor habitat suitability for vulnerable wildlife populations is important for efficient conservation planning (Turner et al., 2003; Turner & Gardner, 2015). Individual species may have multiple habitat requirements which typically differ across spatial scales (Nitschke et al., 2020; Shifley et al., 2006). As such, species‐specific adaptations to a certain climate regime (e.g., fur length) may be determined at a relatively broad scale with low‐resolution data (Turner & Gardner, 2015), while certain habitat requirements (e.g., density of tree hollows for nesting) may require high‐resolution data at much finer scales (Turner et al., 2003). This is often the case for arboreal fauna that rely on the occurrence of nesting and feeding resources, which may be influenced by many other environmental factors, including site productivity, disturbance history, forest structure, and tree morphology and physiology (Dearing et al., 2005; Foley et al., 1999; Gibbons & Lindenmayer, 2002; Lindenmayer et al., 2017; Youngentob et al., 2011).

Herbivore foraging decisions are closely related to plant nutritional quality and their distributions and home‐range sizes are often determined by forage quality, which is driven by foliar chemistry (Au et al., 2019; Foley et al., 1999; Martin et al., 2020; Wallis et al., 2012). For arboreal folivores with specialized diets, such as koalas (*Phascolarctos cinereus*), the concentration and digestibility of protein and amino acids are important determinants of nutritional adequacy (Cork & Catling, 1996; Moore et al., 2004, 2010; Wallis et al., 2010). Protein digestibility can be strongly constrained by the actions of tannins, which form insoluble complexes with proteins (Hagerman & Butler, 1978). Protein accounts for most of the nitrogen (N) found in plant tissue, and concentrations of N, and hence protein, can be limiting for herbivores due to its low concentration and poor digestibility in the plant tissue (Degabriel et al., 2008; Kavanagh & Lambert, 1990; Wallis et al., 2012).

The southern greater glider (*Petauroides volans*, McGregor et al., 2020) is both a threatened species and a folivore whose diet is limited to *Eucalyptus* leaves. Listed as Vulnerable by the IUCN (Burbidge & Woinarski, 2016), it is under threat by anthropogenic disturbances such as climate change and timber harvesting, causing habitat contraction, fragmentation, or loss (Lindenmayer et al., 2011; McLean et al., 2018; Wagner et al., 2020; Youngentob et al., 2013). The greater glider's protein and most of its water intake is obtained exclusively from the foliage of *Eucalyptus* trees (Foley et al., 1990). Such specialization is the rarest form of foraging among mammalian herbivores (Shipley et al., 2009). Home range sizes are 1–4 ha for greater gliders inhabiting mature forests but can increase depending on resource availability (Kavanagh & Wheeler, 2004; Pope et al., 2004; Smith et al., 2007). Factors driving habitat selection and occupancy by greater gliders differ across scales: At a broader extent, a narrow thermal tolerance confines its distribution to the cooler and wetter areas of the landscape (Kearney et al., 2010; McIlwee, 2001; Wagner et al., 2020). At the spatial scale of a forest stand and the greater glider's home range, its specialized diet and the need for mature trees with hollows for nesting together drive habitat selection (Jensen et al., 2015; Kavanagh & Lambert, 1990; Lindenmayer et al., 1990). As such, southern greater gliders are typically found in higher abundance at high elevation and in mature forests that are composed of favored *Eucalyptus* species (Henry, 1984; van der Ree et al., 2004; Youngentob et al., 2011).

In Australia, forests dominated by tree species with average foliar N measures <1% of leaf dry mass (% N DM) are less favorable as habitat for arboreal folivores (Cork, 1992). Greater gliders are known to prefer tree species with high foliar N concentrations (Kavanagh & Lambert, 1990; Youngentob et al., 2011). However, foraging exclusively on *Eucalyptus* foliage is constrained by high levels of plant secondary metabolites (PSMs) that may cause toxicosis or reduce foliage digestibility. This plays an important role in the regulation of feeding and in forage selection by arboreal folivores (Cork & Foley, 1991; Moore & Foley, 2005; Moore et al., 2004; Youngentob et al., 2011). PSMs such as tannins bind to proteins, reducing N digestibility (availability, Marsh et al., 2003), while formylated phloroglucinol compounds (FPCs), are powerful antifeedant defenses against herbivory, that cause herbivores to reject or reduce their intake of defended trees (Lawler et al., 1998; Moore & Foley, 2005). The amount of N in leaves that can be digested (digestible nitrogen, digN) can be a more meaningful measure of forage quality for folivore browsers, especially because N and digN are not always correlated (DeGabriel et al., 2008, 2014). Nevertheless, total N can provide a more general measure of site productivity, often used as a measure of browse quality for the herbivore community in forests (Cork & Catling, 1996; Wallis et al., 2012). Both measures are important to assess feeding habitat: *Eucalyptus* leaves can be high in N and tannins, making these high levels of N unavailable or can be high not only in digN but also in FPCs and, therefore, more resistant to herbivory (Lawler et al., 1998; Marsh et al., 2003; Moore et al., 2004; Wallis et al., 2012). While determining N and digN may only cover parts of what makes a good forage habitat for arboreal folivores (Youngentob et al., 2011), good relationships between leaf reflectance as measured through spectroscopy and these constituents have been reported (Kokaly et al., 2009; Munoz‐Huerta et al., 2013).

Determining foliar nutritional quality of potential habitat trees at the relevant scale presents a challenge. Techniques that require individual leaves, such as chemical analysis and digestion, are impractical due to the enormous number of trees that would need to be sampled and the cost and time involved in processing the resultant leaf samples in the laboratory (Youngentob et al., 2012). Foliar spectroscopy, which uses the light absorption features of chemical bonds in leaves can quantify leaf chemistry at the scale of individual leaves, tree crowns, forest stands, or whole landscapes, depending on the equipment (Ebbers et al., 2002; Kerr & Ostrovsky, 2003; Kokaly et al., 2009). Because the spectral signature of foliage is correlated with foliar chemical properties (Curran, 1989), foliar spectroscopy has proven to be effective for measurements of leaf chemistry and can avoid the challenges imposed by scale. Variations in reflectance can be determined using multi‐ or hyperspectral sensors that are, for example, laboratory based, hand‐held, airborne, or carried by satellites (Kokaly et al., 2009). Like chemical analyses, laboratory‐based or hand‐held sensors are impractical at the scale required for habitat assessments. Most satellite‐based sensors collect data at coarser spatial grains than individual trees or animal home ranges and, therefore, lack the high resolution required to detect variability within leaf or canopy reflectance (Young et al., 2017). For example, current Landsat or Sentinel satellites capture reflectance in 30 × 30 or 20 × 20 m resolution, respectively. Consequently, they cannot provide information on spectral variability at the scale of individual trees, at which arboreal folivore feeding decisions are made (Asner et al., 2017; Attiwill & Adams, 1996; Baldeck et al., 2015; Futuyma & Moreno, 1988; Hume, 1999). High‐resolution alternatives from air‐ or spaceborne hyper‐ and multispectral imagery can be costly, but have been successfully applied in mapping potential favorable feeding habitat for southeastern Australian folivores (Youngentob et al., 2012), as well as koalas in western Queensland (H. Wu et al., 2019) by determining N and digN from spectral reflectance. Another promising tool for acquiring very high‐resolution imagery at a fine scale are unoccupied aerial vehicles (UAVs), or drones.

Unoccupied aerial vehicles are a low‐cost alternative to aerial or satellite imagery. They have been applied in the agricultural sector to estimate crop nutrients and the forestry sector to measure structural metrics of trees (Adão et al., 2017; Felderhof & Gillieson, 2014; Mohan et al., 2017; Nevalainen et al., 2017). UAVs are simple to use, cost‐effective, and highly mobile, making them useful for mapping areas at scales of tens to hundreds of hectares at very high resolution. These platforms can carry various sensors, including multi‐ or hyperspectral cameras, LiDAR, or thermal imagers, which increases their utility for ecological research (Anderson & Gaston, 2013). For example, UAVs have been used to detect koalas (Beranek et al., 2020) and map herbivore feeding habitat (Olsoy et al., 2020) using thermal and multispectral imagery, respectively. While hyperspectral sensors fit for UAVs can be costly and may require larger and heavier platforms, newer generation multispectral sensors are a fraction of the cost and can be readily integrated into small commercial UAVs that can be operated without specialist knowledge or certification (see CASA, 2019).

These sensors contain near‐infrared (NIR) and red‐edge bands to capture reflectance >750 nm wavelength that are needed to distinguish absorption features related to foliar N (Curran, 1989). These bands, along with the three visible‐light bands (red, green, blue) are also useful for developing a range of vegetation indices associated with plant productivity and nutrition (Xue & Su, 2017). High correlations have been reported between N levels of plants, plant productivity, and indices derived from spectral reflectance (Coops et al., 2003; Huang et al., 2004; Munoz‐Huerta et al., 2013; Wang & Wei, 2016). These can provide additional metrics alongside direct reflectance for assessing environmental energy availability as a key determinant in wildlife population dynamics and habitat selection (Munoz‐Huerta et al., 2013; Pettorelli et al., 2011). Plant productivity determined remotely by spectral indices has been linked to greater glider abundance and habitat suitability as well: Youngentob et al. (2015) found that animal detection and abundance increased with higher readings of the normalized difference vegetation index (NDVI), putting additional emphasis on the utility of vegetation indices and remote‐sensing spectroscopy to identify high‐quality feeding habitat for *Eucalyptus* folivores.

With a continuing population decline and evidence of habitat contraction into the cooler and wetter areas of their distribution due to recent climate change (Kearney et al., 2010; Smith & Smith, 2020; Wagner et al., 2020), conservation planning for the greater glider is calling for a better understanding of the factors that determine habitat suitability (DELWP, 2019). Research has focused on the impact of fires and timber harvesting on changes in greater glider occurrence (Lindenmayer et al., 2013; Taylor & Lindenmayer, 2019); however, declines occur in areas not impacted by these disturbances as well (Lindenmayer et al., 2011). Understanding the impacts of climate extremes and foliar nutritional value on habitat suitability is key to developing comprehensive conservation strategies for greater gliders and other arboreal folivores. Incorporating methods that can detect potential feeding habitat at finer scales such as the greater glider's home‐range would enhance current management and conservation planning at an operational scale. The ability to map favorable feeding habitat and identify highly nutritious trees will be beneficial for the management and conservation of arboreal folivores that have strict requirements for feeding and nesting resources (Eyre, 2006; Kavanagh & Lambert, 1990; Lindenmayer et al., 2004). In this study, we tested whether high‐resolution multispectral imagery, collected by a lightweight and low‐cost commercial UAV, can be used to model and predict N and digN at the tree canopy level and identify favorable feeding habitat for southern greater gliders at a stand‐ or home range scale (4 ha). We explore the potential for mapping feeding habitat and test for relationships between spatial feeding metrics such as potential favorable feeding area and detectability.

## 2 MATERIALS AND METHODS

### 2.1 Study area

The East Gippsland region in eastern Victoria, Australia, has a high diversity of *Eucalyptus* species and contains considerable areas of greater glider habitat. East Gippsland has ~1.2 million ha of mixed species eucalypt forests (Dept. of Agriculture & Water Resources, 2018), ranging from open lowland forests dominated by *Eucalyptus sieberi*, *E*. *tricarpa*, and *E*. *globoidea* to dense, high‐elevation forests (~1000–1300 m a.s.l.) with *E*. *delegatensis*, *E. viminalis*, *E. cypellocarpa*, *E. croajingolensis*, and other high‐elevation species (Opie et al., 1990; Sebire & Fagg, 2009). *Eucalyptus obliqua* is the most abundant tree species and occurs across the entire elevation gradient (Dept. of Conservation & Natural Resources, 1995). Mean annual rainfall and temperature for the region are 648–1178 mm (1980–2020, Stewart et al., 2020) and 6–15.8°C (1980–2020, Stewart & Nitschke, 2017), respectively. Greater gliders have been recorded across the elevational range and in a variety of forest types, making this area especially suitable to study inter‐ and intraspecific differences in leaf nutritional quality and their influences on greater glider habitat suitability. Greater gliders are most common above 700 m in closed cool and wet forests where their thermoregulatory requirements are consistently met (Bennett et al., 1991; Henry, 1984; van der Ree et al., 2004; Wagner et al., 2020).

### 2.2 Survey design

We selected study sites based on the region's elevational and topographic range (i.e., aspect and slope). Survey plots were located within three elevation bands (lowlands: 0–420 m; mid‐hills: 420–840 m; and high elevation: 840–1260 m a.s.l). In each of these three elevation bands, we established 10 plots (total *N* = 30 plots, Figure S1, Table 1, and Table S1). Within each elevation band we captured variability in topography by establishing four plots in flat or gully floor locations, four in mid‐slope positions (north or south facing), and two plots on ridges (Figure S2). We only selected study sites that had not been subject to timber harvesting or burnt 30–40 years prior to sampling. We collected multispectral imagery using an unoccupied aerial vehicle (UAV) and leaf samples from individual trees in early summer 2018 and 2019. The plot network was designed to cover the range of eucalypts known to act as forage resources for the greater glider, as well as several species not known to be utilized (Comport et al., 1996; Cunningham et al., 2004; Kavanagh & Lambert, 1990).

**TABLE 1:** Description of each study plot

Position	Plot	Elevation (m.a.s.l)	Dominant canopy species	Measured meanN (% DM)	Measured meandigN (% DM)	No. of greater gliders observed	predicted meanN (% DM)	Mean likelihood of finding pixels ≥1%N DM	Fraction of total plot area favorable (%)	Spatial aggregation (clumpiness metric)
Lowlands	1	120	*E. sieberi*	1.01	0.95	0	1.16	0.61	30.28	0.78
	2	181	*E. consideniana*	1.37	0.81	0	1.32	0.63	46.01	0.67
	3	397	*E. sieberi*	1.00	–	0	1.06	0.50	33.97	0.56
	4	222	*E. polyanthemos*	1.00	–	0	1.19	0.47	7.25	0.64
	5	126	*E. globoidea*	0.94	0.88	0	1.00	0.42	14.21	0.68
	6	61	*E. globoidea*	1.01	0.87	0	1.17	0.55	23.73	0.73
	7	159	*E. consideniana*	1.12	0.84	0	1.15	0.56	29.13	0.73
	8	203	*E. consideniana*	0.88	0.81	0	0.99	0.36	13.43	0.46
	9	282	*E. consideniana*	0.89	0.84	0	1.11	0.38	14.86	0.48
	10	32	*E. globoidea*	1.20	0.90	2	1.23	0.61	29.65	0.74
Mid‐hills	1	439	*E. obliqua*	1.14	0.93	0	1.26	0.66	61.40	0.65
	2	661	*E. fastigata*	1.14	0.97	1	1.24	0.88	88.09	0.85
	3	777	*E. cypellocarpa*	1.39	0.92	1	1.30	0.64	25.11	0.78
	4	679	*E. sieberi*	1.29	0.91	0	1.32	0.63	59.72	0.64
	5	501	*E. muelleriana*	1.20	1.10	0	1.27	0.70	73.71	0.52
	6	448	*E. globulus subsp. pseudoglobulus*	1.23	1.15	0	1.22	0.73	60.43	0.78
	7	692	*E. cypellocarpa*	1.49	0.98	0	1.41	0.75	70.78	0.74
	8	710	*E. sieberi*	1.16	1.16	0	1.31	0.86	87.74	0.75
	9	720	*E. fastigata*	1.71	1.13	0	1.56	0.76	79.57	0.60
	10	446	*E. obliqua*	1.44	–	0	1.39	0.82	84.22	0.80
High elevation	1	926	*E. croajingolensis*	1.60	–	2	1.44	0.67	61.61	0.56
	2	1001	*E. croajingolensis*	1.48	–	0	1.44	0.68	63.51	0.64
	3	1185	*E. delegatensis*	1.58	–	0	1.45	0.57	51.49	0.66
	4	1050	*E. nitens*	1.47	–	0	1.42	0.74	39.20	0.87
	5	907	*E. viminalis*	1.15	–	2	1.25	0.88	64.77	0.94
	6	937	*E. croajingolensis*	1.59	0.88	14	1.41	0.76	64.42	0.76
	7	1072	*E. cypellocarpa*	1.75	1.12	3	1.52	0.71	65.53	0.68
	8	920	*E. obliqua*	1.33	1.01	0	1.34	0.78	73.74	0.72
	9	1117	*E. obliqua*	1.35	0.95	3	1.24	0.50	37.64	0.58
	10	924	*E. viminalis*	1.67	1.20	5	1.54	0.64	57.33	0.60

#### 2.2.1 Forest structure

The position of each plot center was recorded to an accuracy of ~5 m with a handheld GPS (Garmin, Olathe, USA) using waypoint averaging for a minimum of 90 min per plot. In each plot, we recorded elevation, slope, aspect, crown cover, and basal area of the dominant tree species. Basal area was measured using a Kramer's dendrometer and a basal area factor (BAF) of four. This variable radius approach ensured the sampling would capture the large, mature, and dominant trees that form the canopy, and which are preferred nesting and feeding trees for greater gliders that may occupy sites (Kavanagh & Lambert, 1990). The largest plot size resulting from this method was 4 ha, which is congruent with the home range size of greater gliders in mature forests (Lindenmayer et al., 2004; Pope et al., 2004). For trees within BAF = 4, we recorded the species, diameter at breast height (1.3 m, DBH), height, crown widths (both measured using a Vertex IV, Haglof, Långsele, Sweden), estimated tree health, and counted the number of hollows using binoculars. This information was used for another aspect of this study (see Wagner, 2021). All other vegetation (i.e., ground‐level vegetation) were recorded for presence only in a 100‐m^2^ subplot around the plot center.

#### 2.2.2 Sample collection

Leaf samples of mature leaves were collected from five dominant trees per plot. The trees were selected to be tall mature *Eucalyptus*, to be later identified in aerial UAV imagery. One tree was sampled in each quadrant of the plot (i.e., northeast, southeast, southwest, northwest) with an additional dominant tree selected randomly (Figure S3). These five sample trees were selected to capture both the mixture (number of trees) and dimensions (diameter and height distribution) of *Eucalyptus* species in the plot. Leaf samples were collected using a throw‐line launcher and rope, creating a sling around a branch to break it (see Youngentob et al., 2016 for details). A total of 150 trees were sampled, with ~100 g fresh leaf sample material collected from each tree. Individual samples were put in an airtight zip lock bag and frozen after collection. The location of each sampled tree was recorded using GPS waypoint averaging for a minimum of 30 min, as well as bearing and distance from the plot center to later georeference sample trees in the aerial UAV imagery. Leaf samples were measured for average Normalized Difference Vegetation Index (NDVI) using a handheld GreenSeeker crop sensor (Trimble) on a black tarp after collection. Index values (Table S1) were used as a mask before pixel extraction from multispectral imagery to distinguish leaves from other parts of the crowns (e.g., branches).

#### 2.2.3 Capturing canopy reflectance

We collected aerial multispectral imagery for an area of ~20 ha around each plot center using an autopiloted Phantom 4 Pro V2 multirotor UAV (DJI, Shenzhen, China), modified to carry a Rededge‐M multispectral sensor (MicaSense, Seattle, USA, Figure S4). The sensor captures data on five separate spectral bands (red, green, blue, red‐edge and near‐infrared [NIR]) at center wavelengths 475, 560, 668, 717, and 840 nm, respectively. This range is associated with leaf absorption features for chlorophyll a and b, lignin, and protein (Curran, 1989; Curran et al., 2001; Ferwerda et al., 2006; Huang et al., 2004; Peñuelas et al., 1994). Flight paths were preprogrammed using Ground Station Pro (DJI, Shenzhen, China) and planned in accordance with Australian Civil Aviation Safety Authority regulations at a maximum flight altitude of 120 m above ground and within visible line of sight (CASA, 2019). Due to the homogenous canopy structure in many plots, an image front‐ and side‐overlap ratio of 90% was chosen and flights were executed between two hours before and after solar equilibrium of the survey day to avoid shadows and ensure consistent ambient light conditions in all images (Dandois et al., 2015). To accurately map ground elevation, the UAV was launched from canopy openings or forest roads, which ensured sufficient reference ground imagery was captured for later processing. The aerial imagery had a spatial resolution of ~2.5 cm.

#### 2.2.4 Wildlife surveys

Spotlighting surveys on 1‐km transects along a forest track adjacent to, or (where possible) through the plot center, were undertaken at all plots. An average pace of 10 min per 100 m was used by two observers walking 10 minutes apart to maximize the probability of detection (Kissling & Garton, 2006; Nelson et al., 2018). The locations of all arboreal fauna detected were estimated from distance and bearing to the observer position. In addition, we collected data on tree species on which an animal was observed, height in tree, behavior (e.g., feeding or not feeding), color morph, and time of observation.

### 2.3 Sample and data processing

#### 2.3.1 In vitro chemical analyses

Collected fresh leaves were frozen after collection and later dried using a VirTis BenchTop Pro freeze dryer (SP Industries, Stone Ridge, USA) for 24–72 h. Dried samples were ground to pass a 1‐mm screen using a Cyclotec 1093 sample mill (Foss, Hilleroed, Denmark) and kept frozen at −20°C until further analysis. Total nitrogen (N) was determined using combustion based on the Dumas procedure in a TruMac CN analyzer (Leco, Castle Hill, Australia). Digestible nitrogen (digN) was quantified through in vitro digestion using cellulose and pepsin according to Degabriel et al. (2008). Briefly, duplicate 0.5‐g samples were weighed into F57 fiber filter bags (Ankom, Macedon, USA) and sealed, before sequential digestion in buffer, pepsin, and cellulase over 5 days. Residues were weighed and residual N content determined as for N. Both N and digN were reported as % dry matter (% DM).

#### 2.3.2 Multispectral imagery processing

**FIGURE 1:**
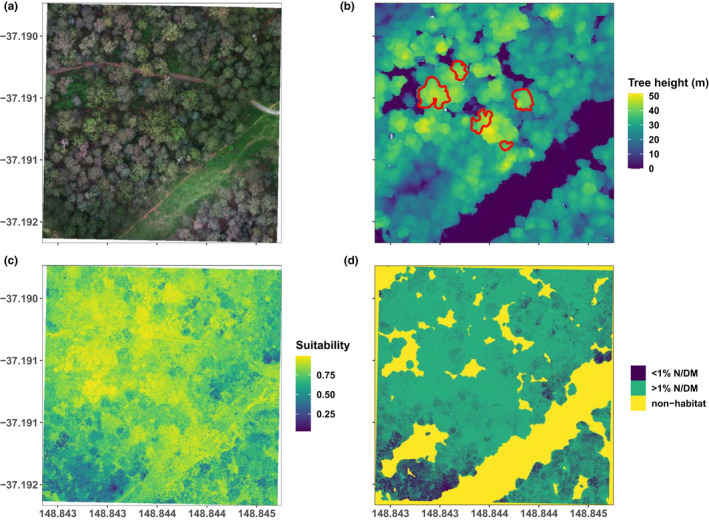
Examples of UAV imagery data products and spatial model prediction workflow. (a) True‐color (RGB) orthomosaic of a plot center (High elevation—Plot 6) and surrounding forest area (4 ha). (b) Final canopy height model of the same plot with sample‐tree crown outlines in red. These crowns were detected using the point‐cloud and CHM and corrected manually using aerial interpretation. (c) Raw spatial predictions from the supervised classification RF model to the sample area of 4 ha indicating likelihood of finding >1% N DM in a pixel from 0 to 1, where 1 is 100% likelihood (suitability). (d) Finalized spatial predictions after removing pixels not associated with the forest canopy using the CHM and minimum subcanopy heights measured as a mask, as well as classifying spatial predictions from percent suitability to the presence and absence of a favorable feeding habitat (≥1% N DM) based on Cohen's kappa (maximum true‐positive and true‐negative rate)

Unoccupied aerial vehicle multispectral imagery was processed in Metashape (Agisoft, St. Petersburg, Russia) using Structure from Motion (SfM), a technique of photogrammetric range imaging to estimate 3D structure from 2D overlapping image sequences (Ullman, 1979). First, all images were calibrated from raw pixel values to absolute spectral radiances for the day of image acquisition using images collected from a reflectance panel exposed to direct sunlight, recorded before and after each flight. The processing in Metashape produced 3D point clouds and georeferenced, multispectral stitched images of all photos (orthomosaics) for each plot and its surroundings (Figure 1a). Orthomosaics were exported as multilayer raster stacks with one band for each wavelength, while point clouds were used to compute a canopy height model (CHM) of each plot. All spatial processing was carried out in R (R Core Development Team, 2020) using the packages *raster*, *sf*, and *lidR* and their dependencies (Hijmans, 2019; Pebesma, 2018; Roussel & Auty, 2019).

#### 2.3.3 Canopy height models and tree detection

To create CHMs for each plot, 3D point clouds were normalized to a 0‐m ground elevation. During processing, a digital terrain model (DTM) and a digital surface model (DSM) were calculated, expressing both the ground elevation and point elevation above ground. The CHM is the difference between DSM and DTM (CHM=DSM‐DTM) and extracted as a 2D raster, expressing each raster cells height above ground in meters (Figure 1b). These were derived using *lidR*’s grid_canopy function with a resolution of 0.5 m and a subcircle algorithm using a 0.5‐m disk on each point return to close empty cells (pits) in the final output (Khosravipour et al., 2014). A tree detection algorithm was applied to each CHM, informed with a fitted function of tree height and mean crown width, derived from field measurements (see Popescu & Wynne, 2004), to approximate the position of each dominant tree in the area covered by UAV multispectral imagery around the plot center. Approximate crown outlines for each detected tree were then extracted using a tree segmentation algorithm based on marker‐controlled watershed object detection, using the approximate tree positions extracted earlier as markers (Gaetano et al., 2014). These approximate crown outlines were used to aid in delineating sample trees from the orthomosaics for crown pixel reflectance extraction (Figure 1c). Using the GPS positions of plot centers and sample trees, we cross‐referenced both CHM and orthomosaics to confirm ground accuracy of <1 m.

#### 2.3.4 Index calculation

To test the performance of UAV multispectral imagery in predicting measures of foliar nitrogen, we used direct reflectance (radiance band values), spectral indices, and simple ratios derived from the five initial bands as predictor variable in a multivariate model. Indices matching available bands and wavelengths were chosen from studies testing different spectral bands and indices as predictors of plant chemistry (see Chen, 1996; Pastor‐Guzman et al., 2015; Xue & Su, 2017; Wu et al., 2019). We derived a total of 14 indices using band calculation on raster stacks of each plot's orthomosaic (Table S2). Together with the 5 initial spectral bands, a total of 19 variables were considered.

### 2.4 Canopy nitrogen data analysis

#### 2.4.1 Extracting reflectance and index values for sample trees

The accuracy of tree positions and crowns from CHM delineation was assessed visually using true‐ and false‐color composites of plot orthomosaics. In cases where sample trees were not correctly delineated (~10% of all trees), the position and crown shape were corrected manually using image interpretation and distance and bearing from the plot center, aided by the structural measurements collected in the field (e.g., height or crown width). From confirmed crown outlines, a vector polygon layer was created, covering the crown of each sample tree as extraction masks. Using *raster's* extract function, we extracted all crown pixel values from the 19 band raster stacks of canopy reflectance and indices using the tree crown polygons (*n* = 150). To create a canopy dataset, we removed pixels not associated with foliage (e.g., branches or bare ground) by applying a NDVI mask using the minimum NDVI field value of each foliage sample, measured with the crop sensor (Table S1). We then subsampled 1000 random pixels (pixel size = 2.5 cm^2^) from each tree and calculated the mean value of each variable (wavelengths and indices) from the extracted pixels. This resulted in a dataset containing each tree's mean crown reflectance (*n* = 150) of five multispectral bands and the mean value for each of the 14 indices.

#### 2.4.2 Modelling total and digestible nitrogen

We used Random Forests (RF) to model canopy N and digN from spectral reflectance and indices. We chose this machine‐learning method as it is commonly used for predicting leaf chemistry from multispectrakal imagery in different vegetation types. RF accounts for interactions and nonlinearities, and produces reproducible outcomes (see Abdel‐Rahman et al., 2013; Breiman, 2001; Li et al., 2014; Ramoelo et al., 2015). RF modelling and model evaluation were carried out in R using the packages *randomForest* and *caret* (Liaw & Wiener, 2002; Kuhn, 2008). We reduced the number of initial predictor variables to eight by removing highly correlated pairs (Pearson's correlation coefficient *r* ≥ |.8|). We used a stratified random sampling design to ensure data across the entire range of variable values are covered in training and testing datasets. The data were separated into 70% of total data (training dataset) and 30% of total data (validation/testing dataset), by randomly choosing datapoints covering the range of measured N and digN values using the function *createDataPartition* from *caret*. To find the best combination of variables, we tested multiple models on combinations of the predictor variables and compared mean squared residuals and percent variance explained. The optimal number of decision trees was chosen by minimum error rate. To increase model performance, only variables that contributed ≥5% increase in mean square error (IncMSE) if removed, were considered. Models were built on training data (70%) only and performance was tested using cross‐ and independent validation. We evaluated the predictive performance of the models by calculating the *R* ^2^, root mean squared error (RMSE), mean absolute error (MAE), and correlation (*p*) between observed and predicted N and digN values. As model predictive performance varied depending on the random data split, we created 100 independent models based on 100 random splits. We evaluated the standard deviation (SD), standard error (SE), and coefficient of variation (CV), as well as mean and median of *R* ^2^, RMSE, and MAE among all models for both cross‐ and independent validation. The best models were chosen based on the highest *R^2^* and the lowest RMSE to report maximum predictive performance of canopy N from canopy leaf reflectance and vegetation indices. Removing outliers (through interpreting data visualization) assured that respective performances were not deviating markedly from other observations.

### 2.5 Home‐range scale data analysis

We built models to predict spatial patterns of high concentrations of foliar nitrogen and favorable feeding habitat at home range scale (4 ha) using a supervised classification RF model based on a predefined N threshold (1% N DM). For this, we used the same predictor variables we identified to be important in predicting canopy N.

#### 2.5.1 Data extraction and compilation

For spatial predictions of foliar nitrogen, we used the sampled 1000 pixels per sample tree (*N* = 150,000 pixels, pixel size = 2.5 cm^2^) in the supervised classification model. Pixel values were classified into favorable (≥1% N DM) and unfavorable for folivore feeding (<1% DM), according to Cork (1992), based on the respective tree's estimated canopy N. We subsampled pixels based on the ratio of suitable to unsuitable points in the dataset (4:1, 25,000 pixels each) to ensure a balanced sample and reduce the chance of biases that may result in model overfitting (Liu et al., 2005; McPherson et al., 2004). The final plot‐level dataset contained 50,000 value combinations of all bands and indices as predictor input for a binomial response variable describing concentrations of foliar nitrogen based on the nitrogen threshold (0 = <1% N DM, 1 = ≥1% N DM).

#### 2.5.2 Modelling favorable feeding habitat

The supervised classification dataset was also split into training and validation data (70:30%) and a binomial RF built on training data only. Model performance was assessed using accuracy, area under the curve (AUC), sensitivity, specificity, and the true skill statistic (TSS, see Allouche et al., 2006) of the independent validation (on 15,600 pixels). Cohen's kappa was used to determine the threshold distinguishing favorable from unfavorable feeding habitat. The value represents maximum model fit and was used to accurately classify spatial predictions into two classes (Elith et al., 2006, 2008).

#### 2.5.3 Mapping favorable feeding habitat

To analyze the spatial distribution and configuration of favorable and unfavorable habitat based on N, we first cropped raster stacks for each plot to an equal area to enable inter‐site comparisons. A square 4‐ha area around each plot center was chosen. This aligned with the maximum recorded plot area in the field based on the variable radius sampling using a BAF = 4 prism and average documented greater glider home range sizes in mature forests (Pope et al., 2004). We used the binomial RF model to predict the probability of finding favorable feeding habitat in each pixel of the cropped raster stacks (Figure 1c). As foliage sample collections were restricted to the upper canopy in order to ensure compatibility with the multispectral UAV imagery, we excluded all pixels below the minimum subcanopy height measured in the field (Table S1). We used each plot's CHM and field canopy height measures to crop the prediction rasters to the tree canopy layer and excluded all pixels that represented understory vegetation or ground and other non‐canopy features. We calculated the mean and SD of predicted likelihood of a pixel being associated with ≥1% N DM for each plot. To assess the proportion of favorable feeding habitat, we classified each raster into areas of ≥1% N DM or <1% N DM, using Cohen's kappa (maximum sensitivity and specificity). We assigned a separate class (non‐habitat) from previously removed pixels of non‐canopy area to ensure intersite comparability (Figure 1d). We calculated the area and proportion of total area of each class and derived clumpiness, a measure of connectivity and spatial aggregation, using the package *landscapemetrics* (Hesselbarth et al., 2019). These metrics were used to compare plot‐specific configurations of favorable feeding habitat with structural measurements, canopy N and digN measurements and to test for relationships with greater glider detection and abundance using linear models. For this, we also extrapolated the number of favorable and unfavorable trees from the five sampled trees per plot to one hectare according to Bitterlich sampling, using an expansion factor of 25 (*c*, according to BAF = 4) and the trees diameter (*d*):

$$\text{Represented number of trees}=\frac{10,000}{\pi c^{2}d_{i}^{2}}$$

## 3 RESULTS

### 3.1 Structural assessment and canopy nitrogen

**FIGURE 2:**
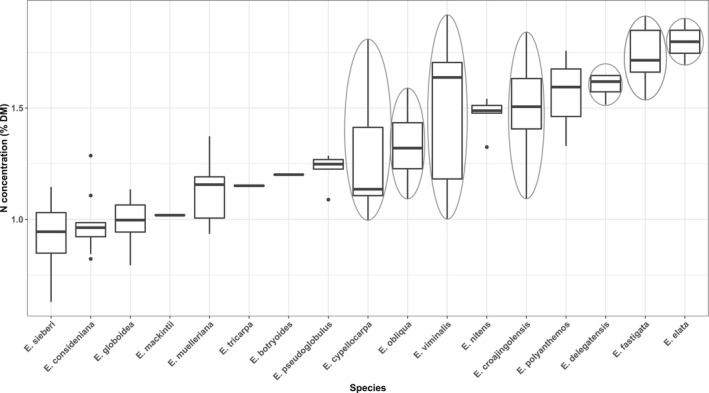
Total nitrogen (N % DM) concentration of the 17 sampled *Eucalyptus* species. Species in which greater gliders were observed are circled in grey. Total number of samples was 150 (*n* = 5 per plot). The number of samples per species can be found in Table S4. *E. pseudoglobulus* *= E. globulus subsp. pseudoglobulus*

Our 30 plots ranged in elevation from 32 to 1185 m a.s.l. and covered slopes from 1 to 55%, as well as aspects from 26 to 355°. Dominant canopy species were *Eucalyptus consideniana*, *E. globoidea*, and *E*. *sieberi* in the lowlands, *E*. *obliqua*, *E*. *cypellocarpa*, and *E*. *fastigata* in the mid‐hills, and *E*. *croajingolensis* and *E*. *viminalis* in the high‐elevation plots (Table 1 and Table S1). Our 150 sample trees covered 17 canopy *Eucalyptus* species. They varied in leaf total nitrogen (N) from 0.63 to 1.92% DM (Figure 2). Mean plot‐level nitrogen (meanN), based on averaging the five sample tree measurements in each plot for a representative sample of the forest structure and canopy species composition, ranged from 0.87% DM in the lowlands to 1.74% DM at high elevation (Table 1). Due to sample loss, we could only analyze 116 samples from 15 *Eucalyptus* species (out of the original 150 samples from 17 species) for digestible nitrogen (digN). Digestions and/or chemical analyses created one true outlier, which was removed from further analyses. DigN ranged from 0.45% to 1.73%DM (Figure S5).

### 3.2 Animal observations

We recorded 44 individual greater gliders while surveying nocturnal arboreal fauna across our plots. Greater gliders were found in 10% of lowland, 20% of mid‐hill, and 60% of high‐elevation plots at varying densities. In the lowlands only two greater gliders were observed in a single plot. In contrast, we found up to 14 individuals during a single survey along a high‐elevation plot (Table 1). Greater gliders were observed in seven *Eucalyptus* species, which were all measured to have leaf N ≥1% DM within our samples (Figure 2).

### 3.3 Evaluating performance of canopy and spatial models

**FIGURE 3:**
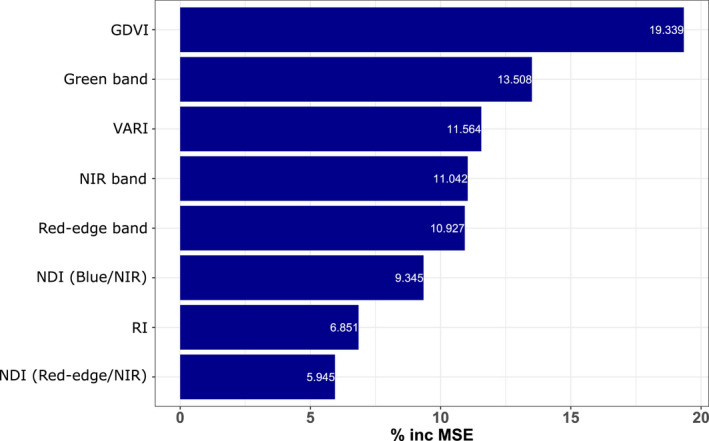
Variable importance plot of the final model of total canopy nitrogen. Variable importance is given as the percentage increase in model mean standard error (% inc MSE) that would occur if the respective variable was removed

All selected predictor variables were found to improve model performance and contribute ≥5% increase in mean square error (incMSE) to the model. Five predictors were indices ($\text{GDVI},\text{VARI},{\text{NDI}}_{\text{B - NIR}},{\text{NDI}}_{\text{RE - NIR}},\text{RI}$, Table 2), while three were direct reflectance averages (green‐, NIR‐, and red‐edge bands). Averaged crown GDVI was the most important predictor with 19.3% incMSE. Overall, indices contributed more to model performance (~53%) than direct reflectance averages (~35%). The most important direct reflectance variable was the green band with 13.5% incMSE (Figure 3).

**TABLE 2:** Spectral indices, their formulae, and literature reference of the five indices that were meaningful in predicting total canopy nitrogen

Index	Full name	Formula	Reference
**GDVI**	Generalized Difference Vegetation Index	NIR‐Green	Wu (2014)
**NDI B/NIR**	Normalized Difference Index ‐Blue/Near Infrared	$\frac{\left(\text{Blue}‐\text{NIR}\right)}{\left(\text{Blue}+\text{NIR}\right)}$	Wu et al. (2019)
**NDI RE/NIR**	Normalized Difference Index Red Edge/Near Infrared	$\frac{\left(\text{Red Edge}‐\text{NIR}\right)}{\left(\text{Red Edge}+\text{NIR}\right)}$	Wu et al. (2019)
**RI**	Redness Index	$\frac{\left(\text{Red}‐\text{Green}\right)}{\left(\text{Red}+\text{Green}\right)}$	Escadafal and Huete (1991)
**VARI**	Visual Atmospheric Resistance Index	$\frac{\left(\text{Green}‐\text{Red}\right)}{\left(\text{Green}+\text{Red}‐\text{Blue}\right)}$	Gitelson et al. (2002)

#### 3.3.1 Best canopy nitrogen model predictions

**FIGURE 4:**
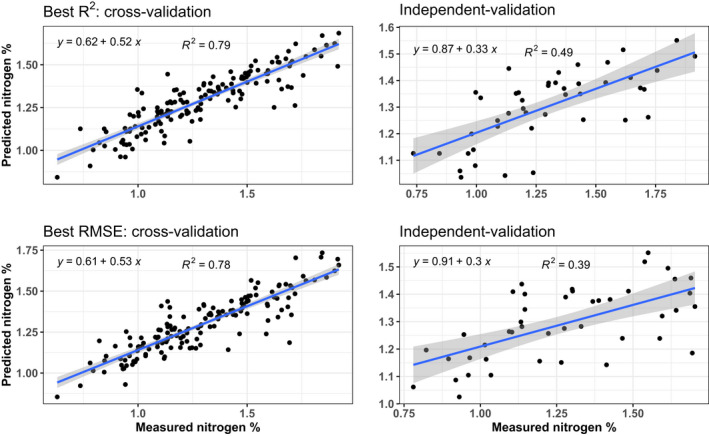
Regression of predicted and observed total canopy nitrogen of the best models based on either *R* ^2^ (top) or RMSE (bottom) for cross‐ (left, *n* = 150) and independent validation (right, *n* = 45)

**FIGURE 5:**
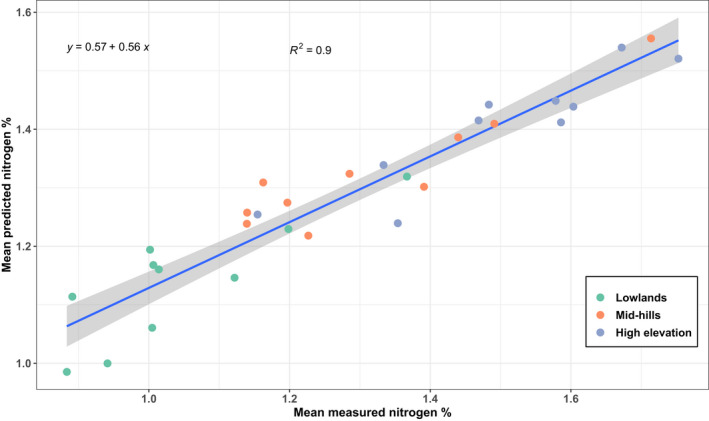
Regression of predicted and observed total nitrogen, averaged on the plot level (*n* = 30)

Canopy‐level predictive performance for both N and digN % DM across 100 independent models is reported in Figure S6, Table 3, and Table S3. Model performance for N was good with mean cross‐validation *R* ^2^ of 0.69 (±0.04). The highest *R* ^2^ model explained 79% of variability between predicted and observed N % DM for all trees (*n* = 150) in cross‐validation and 49% in independent validation (Figure 4). The lowest RMSE was found at 0.15 for cross‐validation and 0.20 for independent validation (Figure S6). For digN, cross‐validation also resulted in 79% of the variability explained, but only 12% in independent validation, indicating that models for N yielded more robust relationships between canopy nitrogen levels and multispectral reflectance and indices than digN. As all selected variables available were used for predicting both N and digN, there was little scope to improve independent model performance for digN without adding additional spectra or developing new spectral indices. Therefore, total nitrogen (N) was used for developing the spatial models with supervised classification. Because the overall independent validation performance was better in the highest *R* ^2^ model (*R* ^2^ = 0.49) at a similar RMSE (0.21), we chose this model for a regression of meanN (mean N of five sampling trees per plot) and the respective average of predictions. This model explained 90% of the variability between predicted and observed meanN % DM (Figure 5).

**TABLE 3:** Average and median value of model evaluation metrics (mean absolute error (MAE), root mean squared error (RMSE), and *r*‐squared from 100 independent models of total nitrogen

Evaluation	Test	Mean	Median	Standard deviation	Standard error
MAE	Cross‐validation	0.128	0.128	0.003	0.00029
	Independent validation	0.210	0.210	0.016	0.00163
RMSE	Cross‐validation	0.169	0.169	0.006	0.00061
	Independent validation	0.256	0.255	0.018	0.00181
*R* ^2^	Cross‐validation	0.687	0.688	0.038	0.00381
	Independent validation	0.203	0.194	0.088	0.00882

#### 3.3.2 Spatial model performance

Both accuracy and area under the curve (AUC) of supervised classification models were 0.78 and 0.88, respectively, with sensitivity (true positive rate, TPR) and specificity (true negative rate, TNR) both at 0.79, resulting in a true‐skill statistic (TSS) of 0.59. This performance indicated substantial agreement between observed and predicted N classes (Table 4). The model correctly predicted 78.6% (6133) of pixels associated with ≥1% N DM and 80.2% (6257) of pixels with <1% N DM (Figure S7). These metrics demonstrated that the spatial model was robust and could be used to map favorable feeding habitat across all plots. Cohen's kappa (maximum TPR+TNR rate) was 0.49 and used as the threshold to classify spatial predictions into areas of <1% and ≥1% N DM.

**TABLE 4:** Model evaluation metrics for the supervised classification model to spatially classify favorable (≥1% N DM) and unfavorable (<1% N DM) feeding habitat

Evaluation metric	Value
Accuracy	0.79
Kappa	0.59
Sensitivity (TPR)	0.79
Specificity (TNR)	0.80
True Skill Statistic (TSS)	0.59
Area under the curve (AUC)	0.88
Correlation	0.67
max TPR+TNR at	0.49
Number of pixels held‐out for validation	15,600
Correctly predicted absences	6257
Correctly predicted presence of favorable feeding habitat	6133
False presences	1543
False absences	1667

### 3.4 Detection thresholds and spatial distribution and aggregation of favorable feeding habitat

An analysis of spatial predictions of N classes and favorable feeding habitat area (Figure 1c,d) at the home range scale identified the likelihood of occurrence (on a scale from 0% to 100%) of pixels predicted to contain ≥1% N DM between 36% and 87%. The lowest likelihood was observed in a lowland plot dominated by *E*. *consideniana* and the highest in a high‐elevation plot dominated by *E. viminalis* and *E. croajingolensis*. The proportion of favorable feeding habitat (areas classified as ≥1% N DM) as a fraction of the total 4‐ha home‐range scale considered here, varied from 7% to 88%, with the largest available feeding habitat area in the mid‐hills, followed by high‐elevation plots (Table 1). Across our plot network, greater gliders were not detected where meanN was <1.1% DM, mean likelihood of ≥1% N DM pixel occurrence was below 50%, and the proportion of favorable feeding habitat was below 25%. We found greater gliders in one‐third of surveyed plots with meanN between 1.1% and 1.4% DM and in two‐thirds with meanN >1.4% DM. Half of all plots in the highest N category had greater glider detections and plots with >25% of the area classified as favorable feeding habitat, detections were made at one‐third of all plots (Figure S8).

**FIGURE 6:**
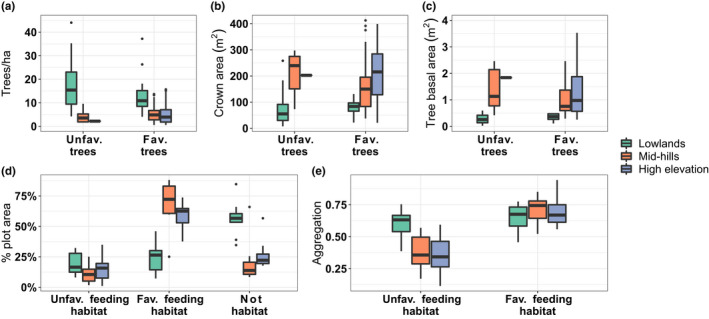
Structural (a–c) and spatial (d+e) differences between favorable (≥1% N DM) and unfavorable (<1% N DM) feeding trees per hectare or habitat (on a home range [4 ha] scale) and elevational bands. Aggregation of nonhabitat was not considered in Figure 6e because it was mostly 100% aggregated through all three elevation bands

Large proportions of the plot area in the lowlands were classified as non‐habitat (e.g., subcanopy crowns or canopy gaps). In addition, unfavorable feeding habitat (areas classified <1% N DM) was most aggregated at the lowland plots. Both favorable (areas classified ≥1% N DM) and unfavorable feeding habitats were on average equally aggregated and covered equal areas in the lowlands, indicating an equal spatial distribution of similarly sized clumps of areas ≥1% and <1% N DM in between large areas of non‐habitat. A high aggregation and larger area of a favorable feeding habitat coupled with a lower aggregation of unfavorable feeding habitat in the mid‐hills and highlands pointed toward larger clumps of interconnected favorable feeding resources, interspersed with small areas of unfavorable habitat (Figure 6d,e). When extrapolating our structural and chemical measurements of the five leaf sample trees from the plot level to a hectare, we observed on average more trees with canopy N ≥1% DM and <1% DM not only in the lowlands but also in the lower crown areas and tree basal area, indicative of a presence of more smaller trees with smaller leaf volume at lower densities. At high elevation there were almost no trees predicted to have <1% canopy N per hectare and crown areas and tree basal areas were highest (Figure 6a–c).

When testing whether N influenced greater glider detection using binomial GLMs, we found a significant relationship between the occurrence of greater gliders and meanN % DM (*p* < .05, Table 5). Mean plot foliar nitrogen concentration was positively associated with a likelihood of greater glider detections (Figure S9).

**TABLE 5:** Model summary for a generalized linear model (GLM) describing the relationship between the detection of greater gliders and mean observed canopy nitrogen by plot

Response	Greater glider detection (GLM)		
Variable	Estimate	95% CI	*p*
(Intercept)	−5.6	−11, −1.0	.03
Mean observed *N*	3.6	0.19, 7.7	.05

Abbreviation: CI, confidence interval.

## 4 DISCUSSION

As remote‐sensing technologies advance, there are many opportunities to use them in spatial ecology and conservation. Here, we show that canopy total nitrogen (N), estimated using a commercial multispectral sensor mounted on an unoccupied aerial vehicle (UAV), can provide accurate high‐resolution data on forest canopy nitrogen, which is useful for identifying favorable feeding habitat for a marsupial arboreal folivore of significant conservation concern. Our approach provides a mechanism for developing spatial models of potential feeding habitat to guide forest management and species conservation planning in forested landscapes.

### 4.1 Leading predictors of canopy nitrogen

While canopy N has been recognized as an important component of the diet of arboreal marsupials and is often determined using laboratory‐based or handheld spectroscopy (Cork & Catling, 1996; Marsh et al., 2014; Moore & Foley, 2005), direct estimates from remote‐sensing platforms have only recently been developed. For example, Youngentob et al. (2012) integrated measures of *Eucalyptus* foliar N and digestible nitrogen (digN) from imaging spectroscopy using aerial hyperspectral imagery, while Wu et al. (2019) showed that digN can be predicted by spaceborne multispectral imagery. Both studies made use of spectral bands and/or vegetation indices derived from these bands, encouraging the application of multispectral imagery in determining eucalypt foliar N as a measure of forage quality. Our eight uncorrelated predictor variables (Figure 3) utilized all available bands and wavelengths covered by our multispectral sensor (475–840 nm). All bands and indices used in our modelling process reflect known nitrogen or associated biochemical absorption features. Three predictors were averaged band reflectances, while five were derived spectral indices. The most important predictor was the Generalized Difference Vegetation Index (GDVI, ~19% increase in mean‐squared error, incMSE), which combines the near‐infrared (NIR) and green band (Table 2). The index covers absorption features that have been detected at 570 nm (green) for chlorophyll and nitrogen (Peñuelas et al., 1994) and 800–900 nm (NIR) for tannins and chlorophyll (Ferwerda et al., 2006). GDVI is also strongly associated with leaf area index (LAI) and shows greater sensitivity to lower vegetation cover than other frequently applied vegetation indices (Wu, 2014). Its high importance in this study suggests that GDVI is capturing crucial absorption features for estimating N, while also being able to distinguish the different vegetation types and associated *Eucalyptus* species we encountered from dry lowland to highly productive high‐elevation sites.

The second most important predictor was the averaged green band reflectance (13.5% incMSE). Green reflectance is known to relate to N absorption features directly, rather than associated biochemicals (Peñuelas et al., 1994). This band is highly sensitive to foliar N levels (Thomas & Oerther, 1972; Xue et al., 2004) and performs better than NIR‐ or red bands (e.g., used in the normalized difference vegetation index, NDVI) at distinguishing vegetation from other features (Gitelson et al., 1996). It may, therefore, combine the ability to directly determine nitrogen through high N sensitivity, while reducing errors arising from background reflectance.

The averaged NIR and red‐edge bands were equally important (~11% incMSE each). NIR is associated with nitrogen between 800 and ~1000 nm (Coops et al., 2003; Curran, 1989), while red‐edge wavelengths at 700–800 nm correlate with chlorophyll and mesophyll absorption, as well as tannins (Curran et al., 2001; Ferwerda et al., 2006; Filella & Peñuelas, 1994). Other important indices, such as the two normalized difference indices (NDI) used here (Table 2), were also found to be meaningful predictors of foliar N in other studies (see, e.g., Wu et al., 2019). Both the Redness Index (RI) and the Visual Atmospheric Resistance Index (VARI) combine the green band's features with other bands associated with chlorophyll (red and blue band) and lignin (blue band) absorption (Curran, 1989; Curran et al., 2001; Ferwerda et al., 2006).

### 4.2 Observed detection thresholds and imperfect detection

We observed higher detection rates for greater gliders with increasing amounts and quality of feeding resources (Figure S8). From canopy N measurements and spatial predictions, we derived three variables describing the quality of favorable feeding habitat: mean plot nitrogen (meanN), likelihood of finding pixels associated with ≥1% N dry mass, DM, and favorable feeding habitat area within a plot (as a proportion of the total area, 4 ha). Greater gliders were detected in plots that had ≥1.1% meanN (DM), ≥50% average likelihood of finding a favorable habitat, and more than a quarter of the plot area as a favorable feeding habitat. The larger crown sizes observed at high elevation (Figure 6b) also indicate a higher foliage volume, while a high aggregation of a favorable feeding habitat (Figure 6e) suggests that this foliage is of higher nutritional quality. Our findings are in agreement with earlier studies demonstrating that greater gliders prefer sites with higher levels of foliar N (see, e.g., Kavanagh & Lambert, 1990). Braithwaite (1983) suggested and Cork (1992) later identified a nutritional threshold of 1% N DM as favorable for arboreal folivore habitat suitability. We found a similar, but slightly higher, nitrogen threshold (1.1% N DM) in our plots. Many studies have found that greater glider occurrence or abundance is higher where *Eucalyptus* species associated with high leaf N contents are present (Braithwaite et al., 1984; Cork & Catling, 1996). We found positive relationships between increasing meanN and detection across our sites (Table 5, Figure S9), providing further support for the importance of foliar nutrition on habitat selection of the greater glider in the study region.

Some plots exceeded all three of the identified thresholds but did not have greater glider detections (*n* = 14, Table 1). While these may be true absence sites, they may also reflect imperfect detection (i.e., gliders were present, but not detected) (MacKenzie et al., 2005). Detection rates for greater gliders at occupied sites are ~66% when surveyed twice or employing two observers per survey (Nelson et al., 2018; Wintle et al., 2005). The observed spatial and structural patterns of potential feeding habitat at different forest types and elevations may explain our detection rates. Although at low elevation the number of both favorable feeding and unfavorable feeding trees were higher when extrapolating from plot measurements to the hectare, both tree basal area and average crown sizes were much lower than at high elevation (Figure 6b,c). At the same time, an equal aggregation of favorable feeding‐ and unfavorable feeding habitat within much larger areas of non‐habitat indicates that foraging resources reaching levels ≥1% N DM were more dispersed and scarcer in the lowlands. Combined with lower tree density and less available feeding resources (i.e., lower crown area, smaller area of favorable feeding habitat), it can be assumed that greater gliders need to move between trees more frequently and travel further distances to reach favorable feeding habitat in suitable areas in the lowlands. The ability to glide allows accessing favorable food resources at further distances while expending little energy (DeGabriel et al., 2009). In fact, the scarcity of nutrients and occurrence of chemical antiherbivory defenses in *Eucalyptus* foliage has been proposed as a factor favoring the evolutionary development of gliding in greater gliders as the most energy‐efficient type of movement between trees for continued feeding and detoxification (Youngentob et al., 2011). Nevertheless, fewer and more dispersed foraging resources may lead to lower population densities in folivores (Chapman et al., 2002, 2004; Wallis et al., 2012). For greater gliders, a combination of smaller areas of more dispersed favorable feeding habitat and lower population densities may require an increase in exploratory movements to reach nitrogen‐rich foliage to forage on or find mating partners and may, therefore, lead to larger home ranges sizes.

**FIGURE 7:**
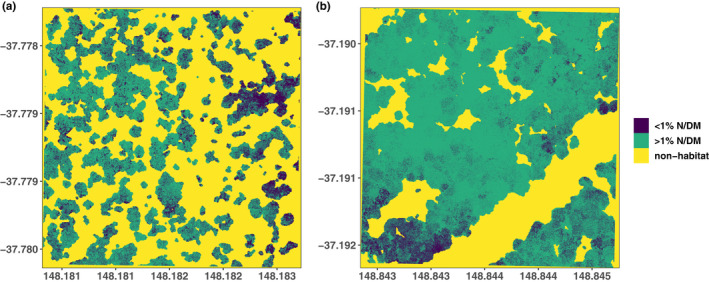
A lowland plot (Plot 10) where two greater glider detections were made outside the plot area (a) and a high‐elevation plot (Plot 6) with three observations within the plot and 14 total observations (b) along a 1‐km spotlighting transect. Both plots have a similar area of unfavorable feeding habitat (<1% N DM in dark blue, ~10% of the plot area) but opposite amounts of favorable feeding habitat (≥1% N DM in green) and non‐habitat (yellow). The lowland plot (a) is predicted to have ~60% non‐habitat (e.g., subcanopy crowns or forest gaps) and ~30% favorable feeding habitat, whereas the highland plot (b) has ~30% non‐habitat and 60% favorable feeding habitat. Assuming the spatial configuration of feeding habitat is constant beyond the plot boundaries, a resident greater glider would require twice as much area in the lowlands to access the same amount of favorable feeding habitat. Assuming average home ranges for greater gliders are constant (e.g., 2.6 ha), a survey through the lowland plot (a) would only cover ~50% of the assumed home range of a potential greater glider occupying the site, while covering an entire home range in the highland plot (b), which has implications on the likelihood of detection

While an increase in home range size due to forage quality has to date not been studied and represents a critical knowledge gap, greater gliders have been observed to increase their home range size due to sparse nesting resources (Pope et al., 2004; Smith et al., 2007; Wagner et al., 2021). We would expect a lower likelihood of detection when using a standardized survey method for a range of forest structures and configurations of feeding habitat or nesting resources. Even when assuming stable home range sizes (e.g., 2.6 ha, see Pope et al., 2004), a survey at a plot with only 30% favorable feeding habitat (≈1.3ha) may only cover 50% of a greater glider's home range occupying the area (Figure 7a). Only when the plot has about ~60% favorable feeding habitat would an entire average home range be covered (Figure 7b, Table 1). At high elevation, structural and spatial patterns such as larger crown sizes almost entirely consisting of favorable feeding habitat (high spatial aggregation and larger areas), lower areas of non‐habitat, and higher basal areas would require less movement and may allow higher population densities and, therefore, smaller home ranges. Consistent with this, we detected up to 14 individuals per survey at high elevation plots, where animals were even observed in the same tree and often in close proximity (Figure 7b). It is therefore likely they had either overlapping or smaller home ranges, supported by a higher abundance of feeding resources. Based on our findings, survey standards may need to be adapted for differences in spatial arrangement of feeding habitat, home range sizes, and population density at different forest types. Our metrics may therefore be a useful guide where non‐detections may require additional surveying efforts to ensure detection and confirm occupancy. Furthermore, the ability to map a potential feeding habitat may help in planning survey transects and increase the likelihood of detection in habitats where foliage quality is limited. Depending on management prescriptions, this tool may then also be applied to protect or retain trees that are important to sustain nutritional suitability and connectivity within the greater glider's home range.

Plant secondary chemistry may influence greater glider foraging, distribution, and, therefore, detection as well. Tannins binding proteins to plant tissue can cause large differences in N and digN in some tree species, while herbivory defenses from formylated phloroglucinol compounds (FPCs) may limit the quality of foliage high in digN (Lawler, Foley, Eschler, et al., 1998; Lawler, Foley, Pass, et al., 1998; Marsh et al., 2003). Some *Eucalyptus* species dominating our sites, such as *E. polyanthemos* or *E*. *muelleriana*, were high in N or digN (see Figure 2 and Figure S5), but we did not make any greater glider observations, which can be explained by other leaf constituents. These species also have high levels of sideroxylonals, which greater gliders do not prefer feeding on (Wallis et al., 2002; Youngentob et al., 2011). The ability to similarly predict and spatially map digN (see Youngentob et al., 2012; Wu et al., 2019) and a method that can remotely sense FPC or unsubstituted B‐ring flavanones concentrations (Marsh et al., 2019) may, therefore, assist in distinguishing and classifying feeding habitat in more detail. To our knowledge, only few such attempts were successful, but the latter may be possible using hyperspectral leaf reflectance (Couture et al., 2016; Ebbers et al., 2002). Given FPCs only occur in one of the two *Eucalyptus* subgenera found in the study area (*Symphomyrtus*), while *Monocalyptus* contain unsubstituted B‐ring flavanones as herbivore deterrents, there may also be potential to use multi‐ or hyperspectral canopy reflectance patterns for classification at the subgeneric level (Baldeck et al., 2015; Goodwin et al., 2005). This may further aid in characterizing the nutritional suitability at the home range scale. Although we accounted for many possible factors that may explain non‐detection or lower population densities, such as fires, drought, or timber harvesting and, therefore, lack of hollows for nesting during site selection, another explanation may be owl predation (see, e.g., Kavanagh, 1988) or historical factors such as disease outbreaks.

### 4.3 Analytical and operational limitations

Both N and digN were significantly associated with the eight spectral predictor variables. Models predicted nitrogen at the canopy level, as well as favorable feeding habitat at the home range scale (4 ha). However, our digN model was found to be explanatory only and lacked predictive power. Predictions exhibited poor performance in independent model validation (best *R* ^2^ = 0.12, average *R* ^2^ = 0.03, Table S3). The reason for this might be that digN is influenced by many constituents, such as N, lignin, tannins, and cellulose concentrations that modify digestibility (Degabriel et al., 2008) and might, therefore, not have a distinct direct relationship to spectral reflectances covered by our sensor or indices. Wu et al. (2019) found that digN was best predicted with blue multispectral bands at lower wavelengths (400–450 nm), while Youngentob et al. (2012) reported the highest predictive performance when integrating at least four hyperspectral bands with wavelengths between ~1300 and 2174 nm. It may, therefore, be the case that our multispectral sensor, which is limited to wavelengths between 475 and 840 nm was unable to register these spectral components of the foliage. Nevertheless, we found that vegetation indices can be used to reduce the limitations small multispectral sensors using center wavelengths may have. Therefore, there can be other indices, not considered here, that may improve predictive performance of digN models, which should be further explored. Indices contributed more to overall model performance than averaged spectral reflectance and Youngentob et al. (2015) also found that spectral indices were useful for predicting greater glider abundance. While hyperspectral sensors may have advantages in terms of spectral range, multispectral imagery is more accessible and affordable, has better cross‐platform integration, and can be easier to process.

When interpolating our canopy models to predict leaf nitrogen spatially at the home range scale, we encountered issues arising from nonlinear averaging (i.e., Jensen's inequality, see Denny (2017)). Canopy models were based on reflectance averages of crown foliage, which had a lower variance than the variance in the predictors at the home range scale. The high variance of the home range scale predictors increased the effect of Jensen's inequality and, therefore, reduced the robustness of average‐condition models for predicting across a wider range of conditions (Roussel & Auty, 2019). We used supervised classification on a binomial response variable describing the presence or absence of a favorable feeding habitat to overcome this issue. Interpolation issues did not arise in similar studies. Youngentob et al. (2012) reported that the use of maximum spectra produced better models than averages, while Wu et al. (2019) used a pixel averaging approach much like ours. We did not find maximum spectra to improve models in our analyses. Our image resolution was much higher (~2.5 cm) than either of the other studies (1.24–7.5 m), due to our UAV imagery being collected from only ~120 m flight altitude. The orders‐of‐magnitude increase in spatial resolution produced greatly improved tree delineation and the quality of our canopy height models (CHMs), which were crucial in classifying spatial predictions and identifying non‐habitat. Nevertheless, it may have exacerbated the effects of Jensen's inequality through the larger number of pixels, which led to greater variance in the raw spectral reflectance than canopy averages of thousands of pixels.

Using small and highly mobile UAVs with spectral sensors to map foliar nutrition quality over large areas has the potential to better integrate forest management and conservation planning. A pragmatic issue that will arise is the type of UAV and the scale of sampling. While our multirotor UAV can readily sample areas of up to 20 ha per flight and, therefore, cover multiple greater glider home ranges, fixed‐wing (FW) UAVs may be better suited to capture imagery over larger areas (Anderson & Gaston, 2013). This will allow for determination of canopy N and feeding habitat at the scale of several square kilometers in a few flights, potentially covering a population‐range scale. However, these platforms require clearings for take‐off and landing, which may not be readily available in heavily dissected or mountainous terrain where, in our case, greater gliders typically occur. In more recent years, FW UAVs have been successfully equipped with vertical take‐off and landing (VTOL) abilities (Goodbody et al., 2017), which may be useful when capturing imagery of larger areas of tall forests in complex terrain.

## 5 CONCLUSION

We used UAV multispectral imagery, individual tree‐sampling of foliar nutrition and forest structure and transect‐based wildlife surveys to successfully characterize and map favorable feeding resources in a landscape of a threatened arboreal folivore, the southern greater glider, in southeastern Australia. Our findings and models have important management implications for the detection and retention of high‐quality feeding habitat at both the canopy level of individual trees and spatially at the home‐range scale, as well as for survey design for arboreal folivores. Although successfully modelling and mapping total nitrogen, digestible nitrogen models could not be extrapolated spatially using the spectral bands and vegetation indices available. The ability to map digestible nitrogen would enhance the classification of folivore habitat in more detail, considering the negative effects of plant secondary metabolites. Future research should focus on identifying additional spectral bands or indices to predict digestible nitrogen spatially. This study is a first step in expanding nutritional studies to larger scales. It is now important to integrate these methods into platforms that can capture larger areas, which will allow a swift and low‐cost assessment of favorable greater glider feeding habitat to aid in their management and conservation at the landscape scale.

## CONFLICT OF INTEREST

The authors have no competing interests to declare.

## AUTHOR CONTRIBUTIONS

**Benjamin Wagner:** Conceptualization (lead); Data curation (lead); Formal analysis (lead); Investigation (lead); Methodology (lead); Project administration (equal); Software (lead); Validation (lead); Visualization (lead); Writing – original draft (lead); Writing – review & editing (lead). **Patrick J. Baker:** Supervision (supporting); Validation (supporting); Writing – original draft (supporting); Writing – review & editing (supporting). **Ben D. Moore:** Methodology (supporting); Resources (supporting); Validation (supporting); Writing – original draft (supporting); Writing – review & editing (supporting). **Craig R. Nitschke:** Conceptualization (supporting); Formal analysis (supporting); Funding acquisition (equal); Investigation (supporting); Methodology (supporting); Supervision (lead); Validation (supporting); Visualization (supporting); Writing – original draft (supporting); Writing – review & editing (supporting).

## Supporting information

Supplementary Material

## Data Availability

The presence and absence data for *Petauroides volans* used in this study and all other animal observations recorded during surveys are available on the Victorian Biodiversity Atlas (https://vba.dse.vic.gov.au/vba/). Due to large file sizes, UAV imagery, point clouds and raster data are available upon request. All other datasets and scripts are available on Dryad via https://doi.org/10.5061/dryad.k0p2ngf9d.
