# Supplementary material for: Mapping canopy nitrogen‐scapes to assess foraging habitat for a vulnerable arboreal folivore in mixed‐species Eucalyptus forests
Source: Ecol Evol. 2021 Dec 16;11(24):18401–21. doi: 10.1002/ece3.8428 (PMC8717341; doi:10.1002/ece3.8428)
Supplement: Supplementary file 1 — Supplementary Material [file ECE3-11-18401-s001.pdf]

Functional Ecology – Research Article – Supplementary Material

Mapping canopy nitrogen-scapes to assess foraging habitat for a vulnerable arboreal folivore in mixed-species *Eucalyptus* forests

Benjamin Wagner[1, *], Patrick J. Baker[1], Ben D. Moore[2], Craig R. Nitschke[1]

[1] School of Ecosystem and Forest Sciences, The University of Melbourne, Richmond, VIC 3121, Australia

[2] Hawkesbury Institute for the Environment, The Western Sydney University, Locked Bag 1797, Penrith, NSW 2751, Australia

*Correspondence: benjamin.wagner@unimelb.edu.au

Supplementary figures

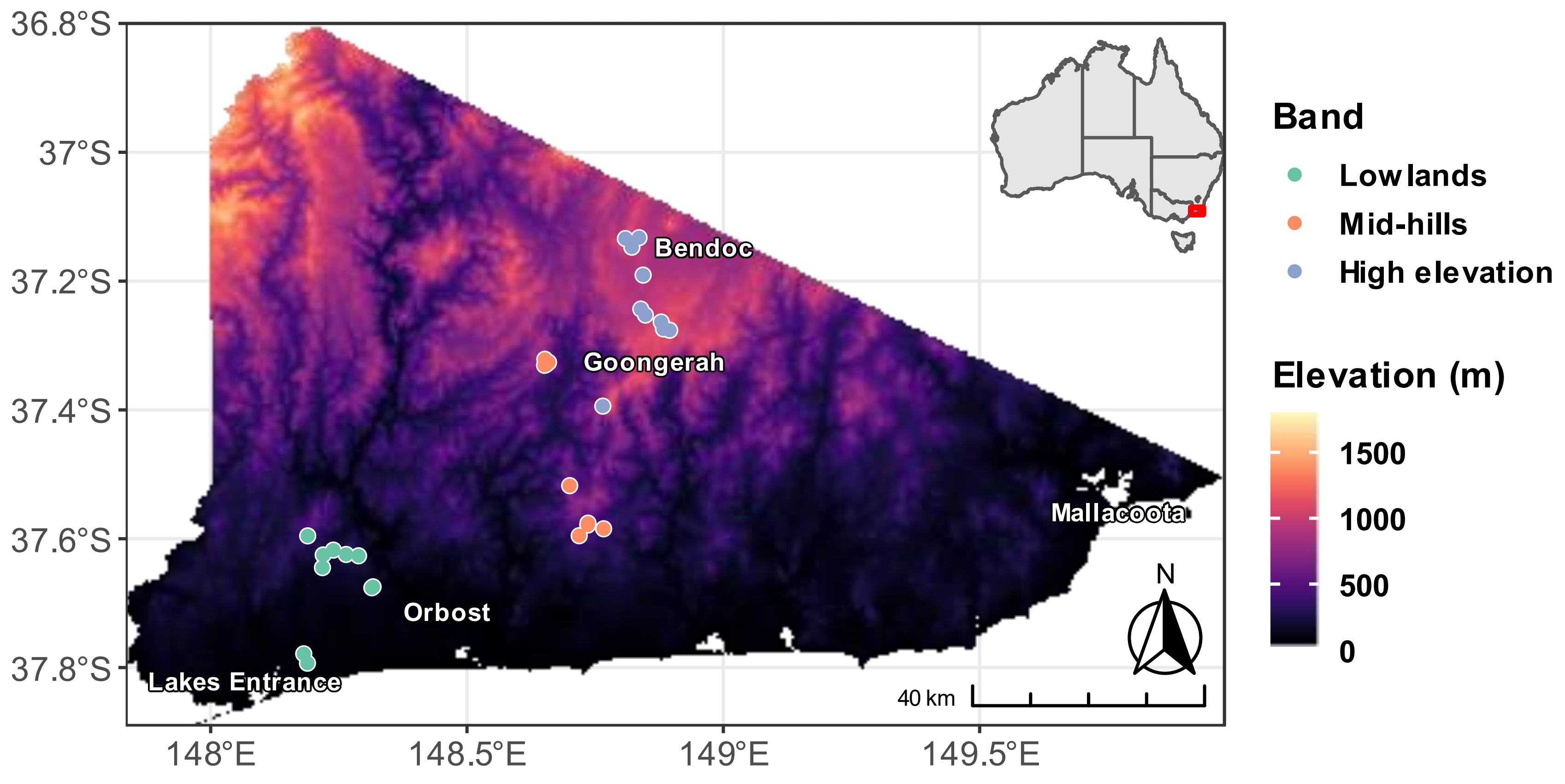

Figure S1. Study area map of East Gippsland, Victoria with major settlements and location of 30 study sites. The colour gradient illustrates elevation above sea level in meters.

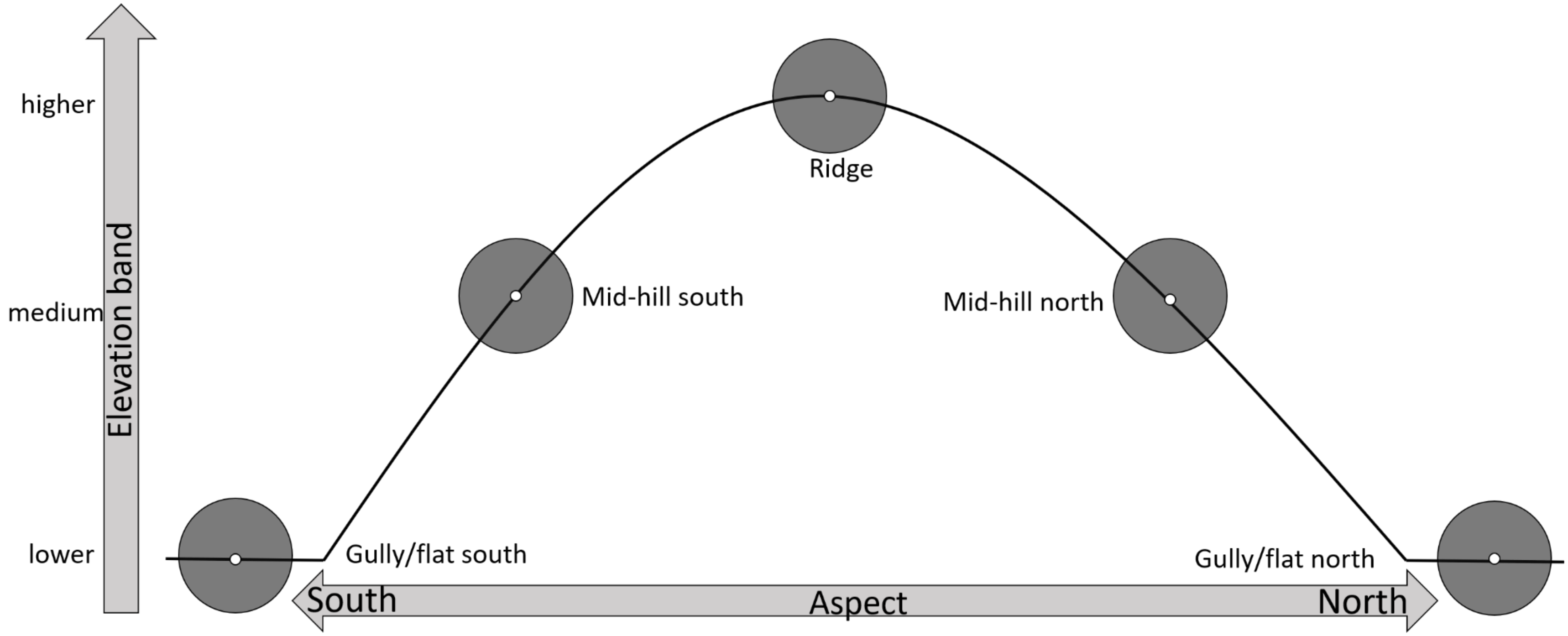

Figure S2. Transect design: Six of these transect were established within three elevation bands (two transects per band) between sea level and ~1200 m.a.s.l. Transects were laid out to ensure capturing the topographic variability of the landscape as expressed by elevation, slope and aspect and through that a variety of dominant Eucalyptus species forming the canopy.

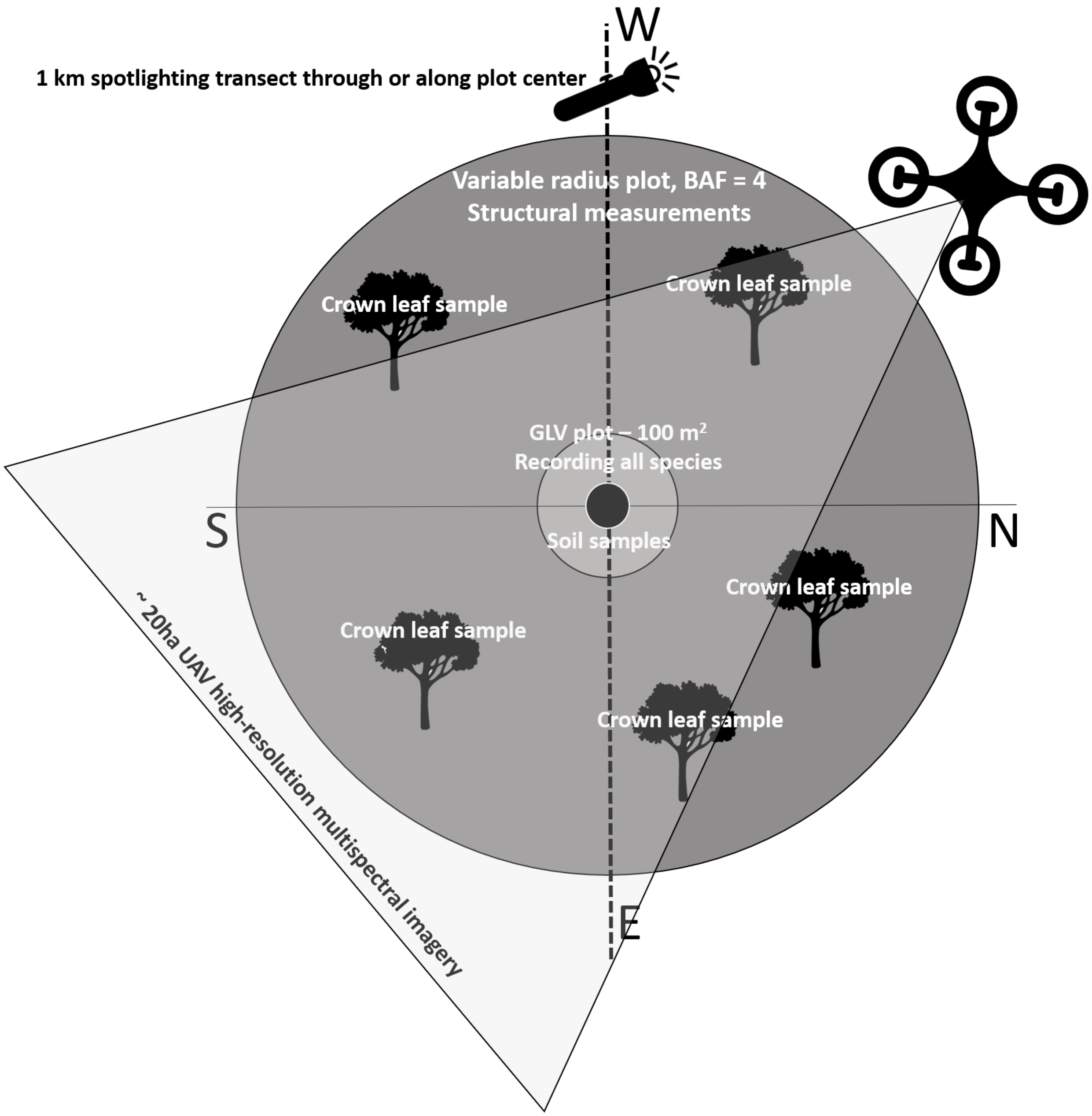

Figure S3. Plot design outlining data collection within each of the 30 sampling plots. All trees falling within the variable radius sample using BAF 4 were measured and leaf samples collection from one dominant tree per plot quadrant and one additional tree selected randomly. Ground level vegetation (GLV) was recorded for species and number of individuals in 100 m^2 sub-plot around the plot center, along with soil samples using a tube corer. At solar equilibrium, multispectral imagery was collected using a UAV over the plot center, covering ~20 ha per site. At night, nocturnal fauna was recorded along a 1 km spotlighting transect.

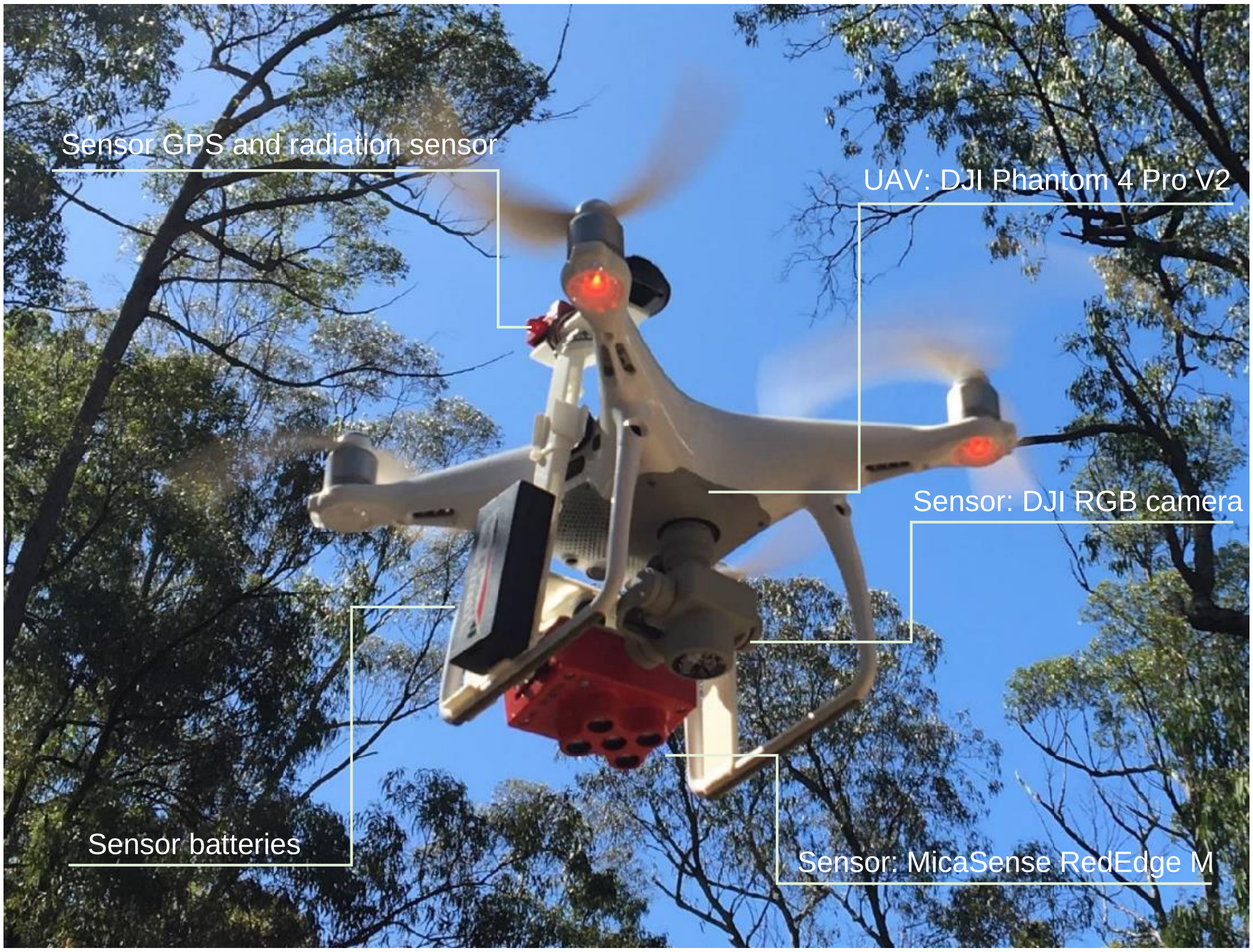

Figure S4. DJI Phantom 4 UAV modified to carry a Micasense RedEdge M multispectral sensor. A 3D printed mount allows to attach the sensor between the landing skids of the UAV. The rod on the left carries the batteries to operate the camera and lifts the radiation sensor and GPS over the propellers for safe operation. This setup allows to capture both multispectral and RGB imagery simultaneously as it leaves the drone sensor operational. The drone sensor can e.g. be used during operation to check for obstacles or ensure the programmed flight-lines are followed correctly.

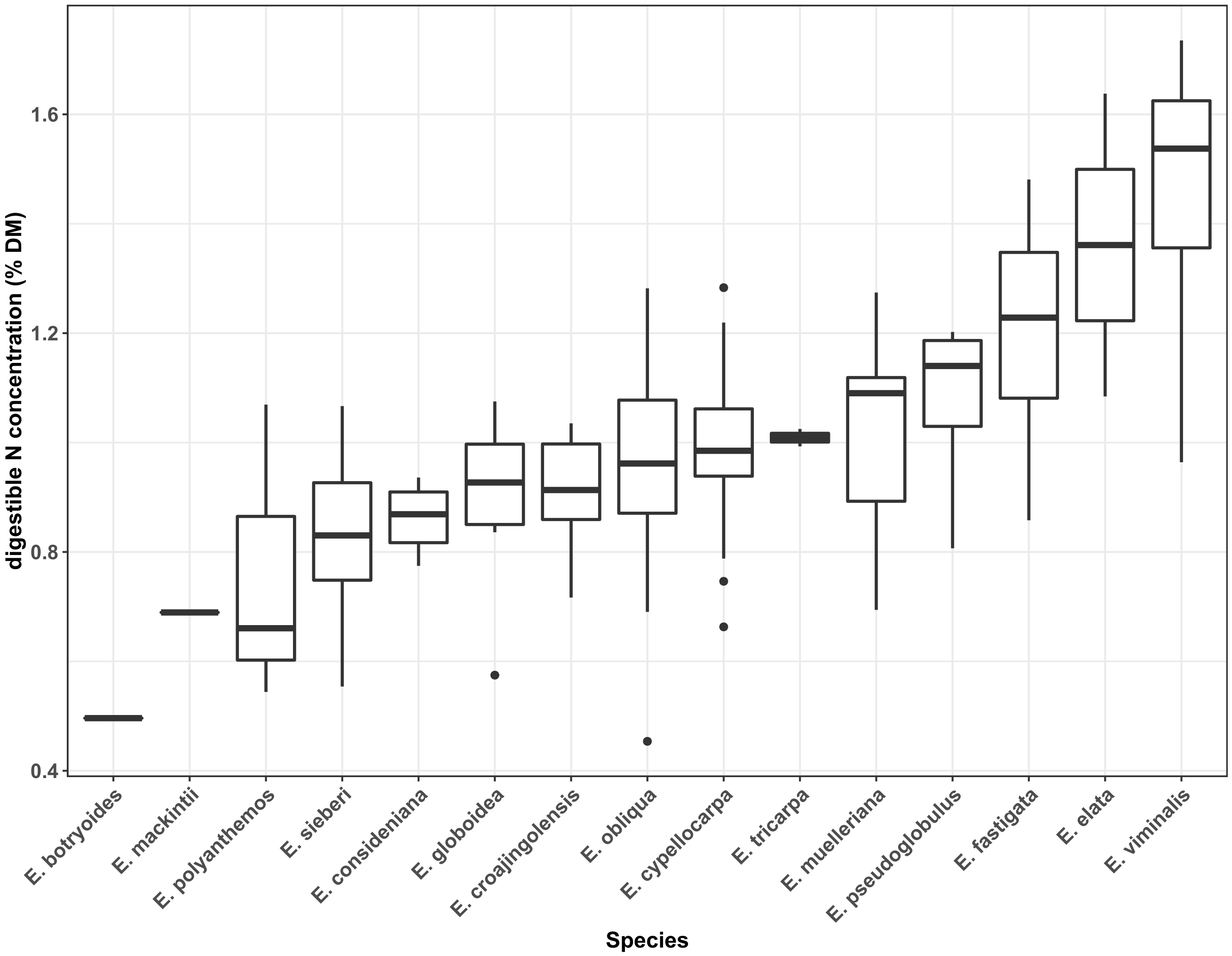

Figure S5. Observed ranges of digestible N (digN) concentration of 15 Eucalyptus species sampled

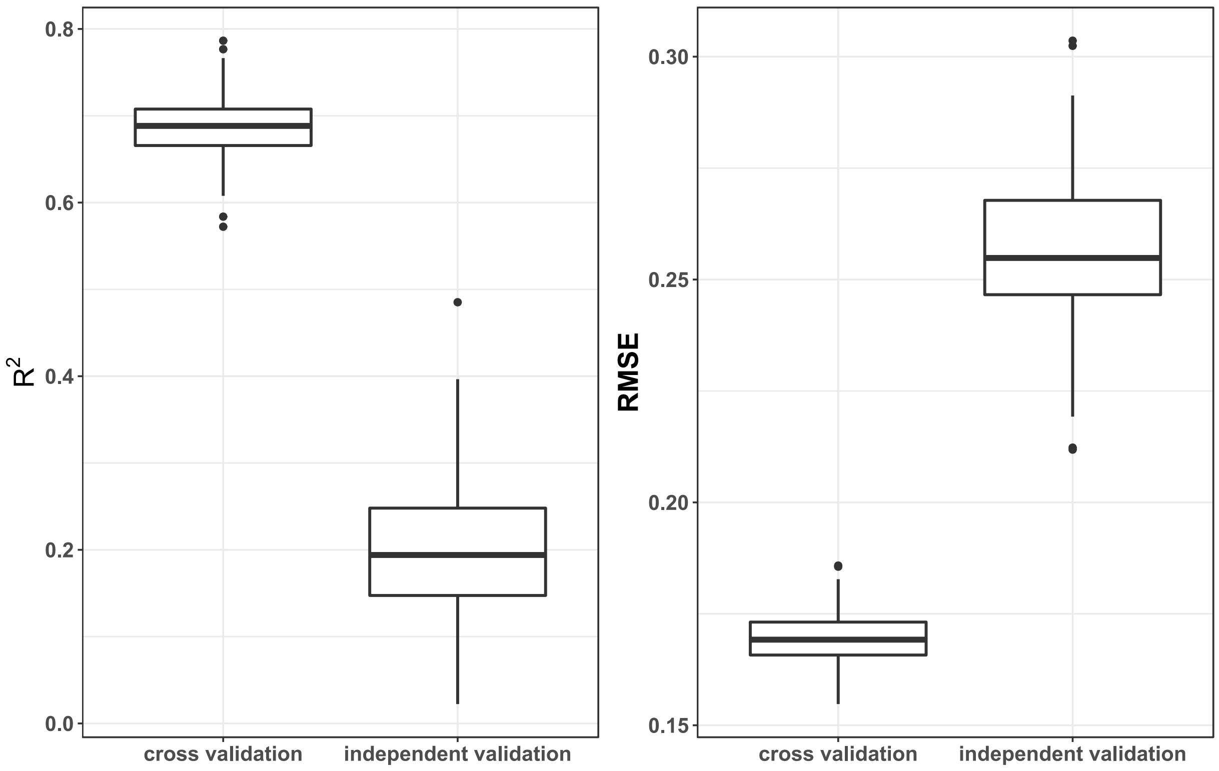

Figure S6. Model performance evaluation of independent 100 models of total nitrogen based on 100 random data splits based on R-squared (left) and root mean square error (right).

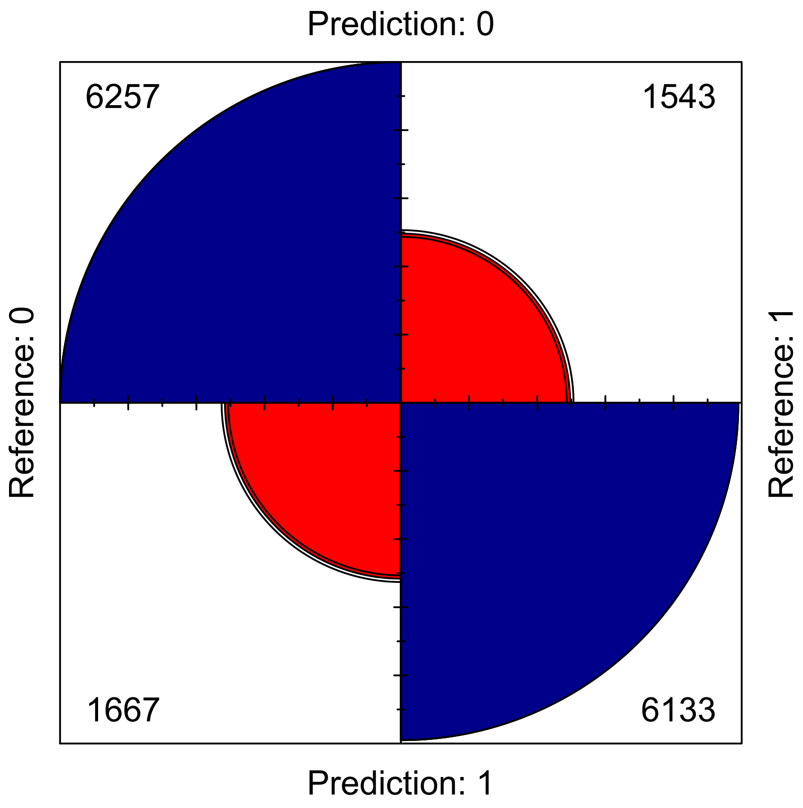

Figure S7. Fourfold plot illustrating independent supervised model prediction accuracy based on the confusion matrix of this model's evaluation. ~80% of pixels associated with unfavourable (top-left) and ~78% of pixels associated with favourable habitat (bottom-right) were predicted correctly (blue charts).

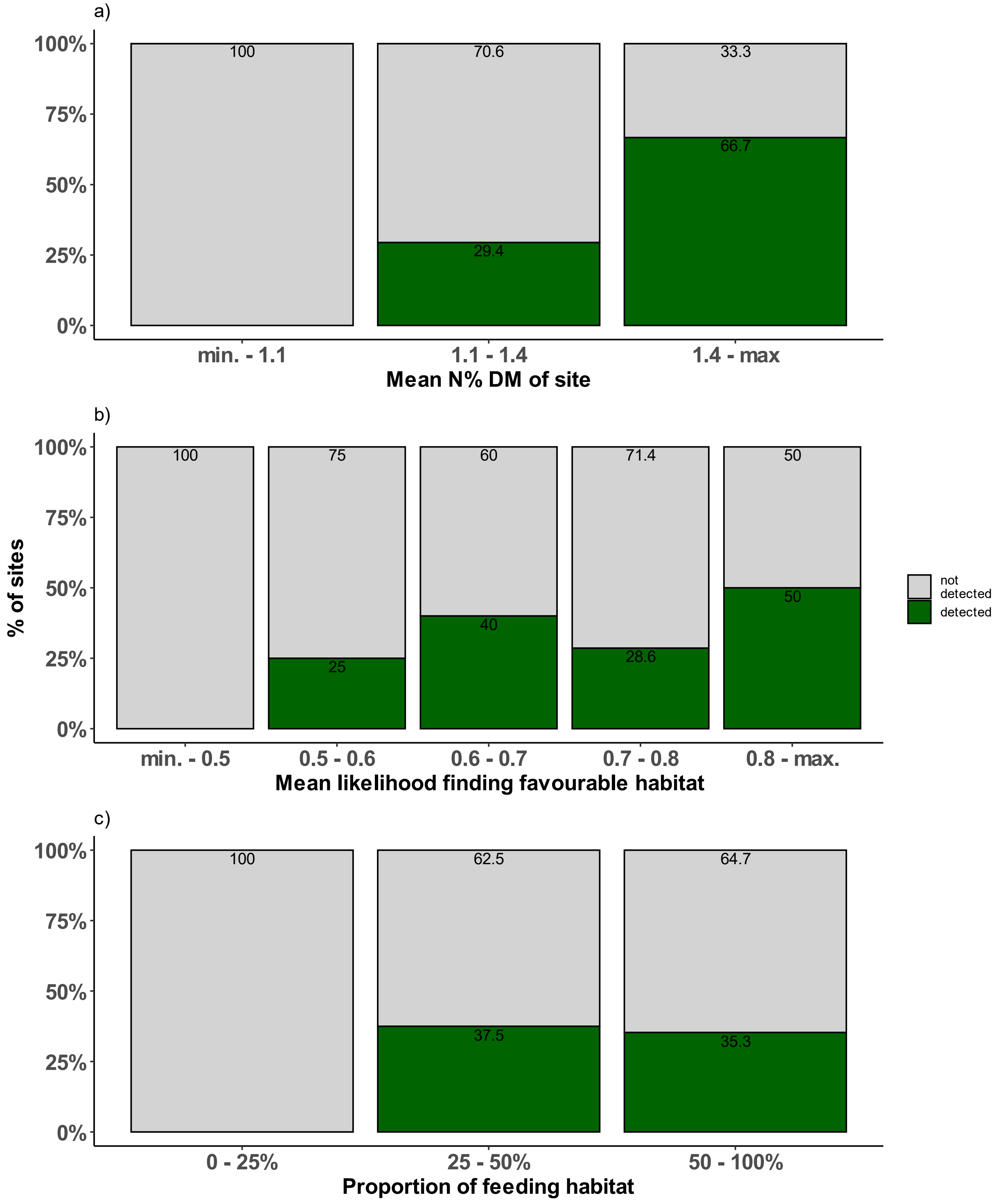

Figure S85. Fraction of occupied sites for the three detection thresholds identified: a) average plot level nitrogen (N) based on chemically determined N measures of five trees per plot, b) mean likelihood of finding pixels associated with favourable habitat (>1% N DM) from the supervised classification model and c) proportion of feeding habitat, i.e. fraction of the site that was classified as > 1% N DM.

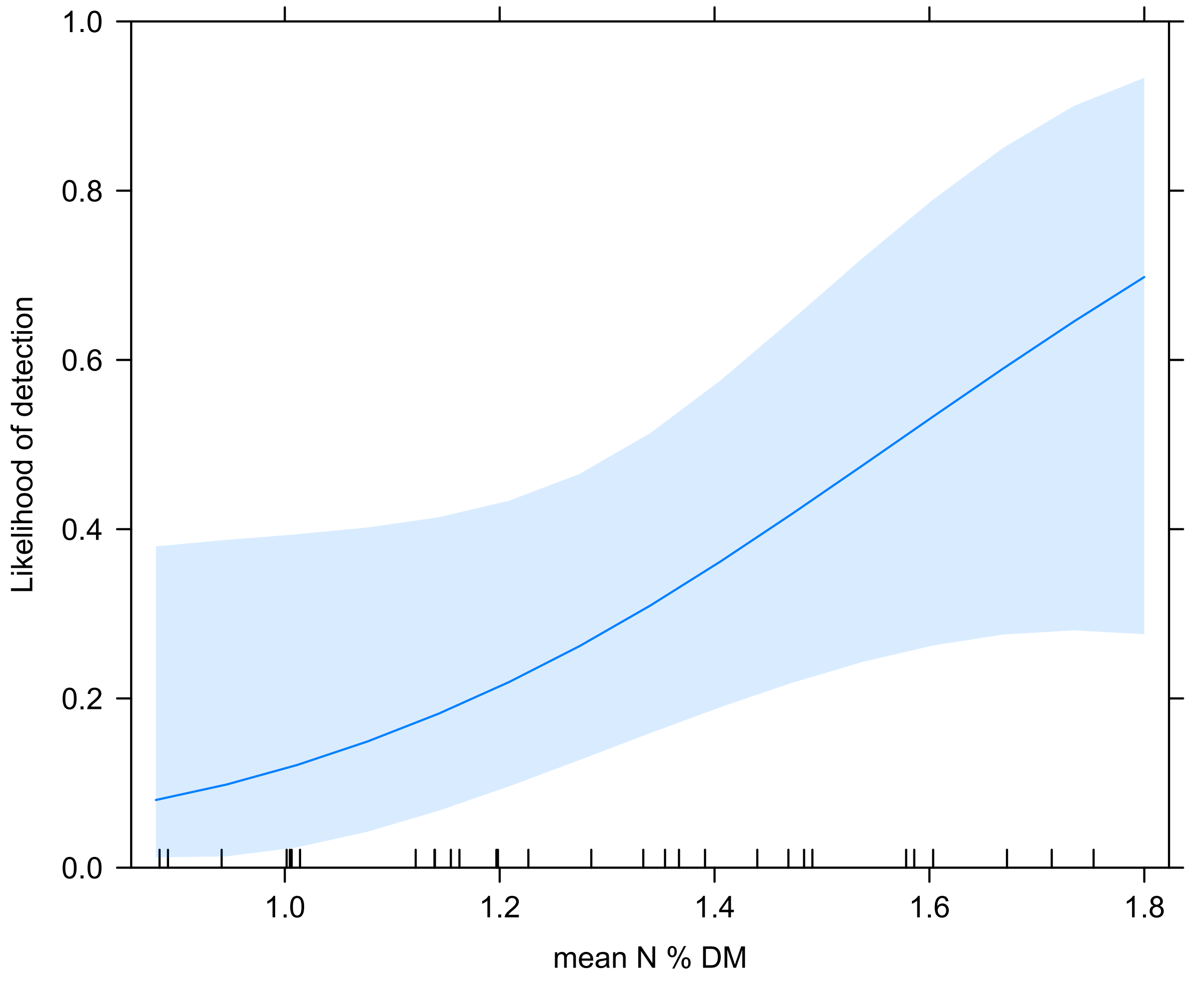

Figure S9. Partial dependence plot for binomial GLM model testing the relationship between mean plot level N and greater glider detection. Increasing levels of total N had a positive effect the likelihood of greater glider detection.

Supplementary tables

Table S1. Site description of the 30 plots assessed for this study

Ground survey date	Transect	Plot	Latitude	Longitude	Elevation (m.a.s.l)	Slope (%)	Aspect (degrees)	Dominant canopy species	Dominant mid/understory species	Mean sample NDVI	Min. subcanopy height (m)
25/09/2018 9:00	T1	1	-37.6747	148.3167	120	3	350	E. sieberi	Acacia mearnsii	0.74	12.7
15/06/2018 10:30	T1	2	-37.6265	148.2884	181	35	180	E. condensiana	E. condensiana	0.74	18.8
14/06/2018 10:45	T1	3	-37.6244	148.2641	397	5	220	E. sieberi	E. sieberi	0.71	8.9
27/09/2018 13:54	T1	4	-37.6176	148.2393	222	23	340	E. polyanthemos	Cassinia longifolia	0.7	13.6
25/09/2018 13:43	T1	5	-37.6761	148.3144	126	2	145	E. globoidea	Acacia longifolia	0.79	13.7
29/09/2018 9:13	T1.5	1	-37.7932	148.1887	61	1	120	E. globoidea	Allocasuarina verticillata	0.73	16.3
26/09/2018 14:55	T1.5	2	-37.6448	148.218	159	8	130	E. consideniana	Cassinia longifolia	0.69	14.8
26/09/2018 10:25	T1.5	3	-37.5955	148.1892	203	1	85	E. consideniana	-	0.63	15.2
27/09/2018 15:51	T1.5	4	-37.6252	148.2194	282	20	355	E. consideniana	Persoonia silvatica	0.72	15.6
29/09/2018 14:10	T1.5	5	-37.7787	148.1815	32	3	60	E. globoidea	Acacia vernicifolia	0.74	20.4
3/10/2018 9:50	T2	1	-37.5953	148.7188	439	10	123	E. obliqua	Cyathea australis	0.87	14
1/11/2018 11:23	T2	2	-37.3261	148.659	661	20	299	E. fastigata	Smilax spp.	0.77	6.5
31/10/2018 11:49	T2	3	-37.3214	148.6518	777	12	93	E. cypellocarpa	Carcinia and Polycyas	0.81	15
30/10/2018 9:14	T2	4	-37.5176	148.7003	679	55	259	E. sieberi	E. sieberi	0.76	4
2/10/2018 9:53	T2	5	-37.5788	148.7356	501	5	300	E. muellerinia	Eleaocarpus reticulatus	0.82	13.7

4/10/2018 9:11	T2.5	1	-37.5845	148.7665	448	3	250	E. pseudoglobulus	Acacia melanoxylon	0.85	23.7
3/11/2018 12:13	T2.5	2	-37.3307	148.6512	692	8	340	E. cypellocarpa	Acacia dealbata	0.78	18
31/10/2018 15:26	T2.5	3	-37.3296	148.6541	710	6	305	E. sieberi	Cassinia longifolia	0.77	16.8
2/11/2018 11:20	T2.5	4	-37.3244	148.655	720	26	50	E. fastigata	Cassinia trinerva	0.79	25
1/10/2018 9:20	T2.5	5	-37.5758	148.7361	446	5	315	E. obliqua	Acacia dealbata	0.77	31.5
11/05/2018 10:47	T3	1	-37.2527	148.848	926	6	26	E. croagingalensis	Persoonia silvatica	0.82	18
9/05/2018 10:03	T3	2	-37.2635	148.8788	1001	14	325	E. croajingalensis	Tasmannia lanceolata	0.83	31
7/05/2018 15:43	T3	3	-37.2743	148.8832	1185	5	345	E. delegatensis	Tasmannia lanceolata	0.78	22.6
8/05/2018 9:26	T3	4	-37.2763	148.8954	1050	10	130	E. nitens	Atherosperma moschatum	0.8	16
10/05/2018 13:39	T3	5	-37.2434	148.8391	907	4	182	E. viminalis	Tasmannia lanceolata	0.76	23.3
6/11/2018 10:20	T3.5	1	-37.1907	148.8435	937	4	330	E. croagingalensis	Caprosma quadrifida	0.75	30
7/11/2018 13:17	T3.5	2	-37.1473	148.8215	1072	20	140	E. cypellocarpa	Acacia dealbata	0.76	12.5
5/11/2018 16:23	T3.5	3	-37.3941	148.7649	920	16	246	E. obliqua	Cyathea australis	0.77	3.5
8/11/2018 11:56	T3.5	4	-37.1339	148.809	1117	17	333	E. obliqua	Leucopogon maccraei	0.8	34.6
6/11/2018 15:18	T3.5	5	-37.1326	148.8356	924	8	49	E. viminalis	Persoonia silvatica	0.76	12.5

Table S2. All spectral indices considered as possible variable candidates in this study

Index	Full name	Formula	Reference
**GDVI**	Generalized Difference Vegetation Index	$NIR - Green$	W. Wu (2014)
**GRVI**	Green Red Vegetation Index	$\frac{NIR}{Green}$	Motohka et al. (2010)
**NDGI**	Normalized differential greenness index	$\frac{(Green - Red)}{(Green + Red)}$	Nedkov (2017)
**NDI B/NIR**	Normalized Difference Index - Blue/Near Infrared	$\frac{(Blue - NIR)}{(Blue + NIR)}$	H. Wu et al. (2019)
**NDI RE/NIR**	Normalized Difference Index Red Edge/Near Infrared	$\frac{(Red\ Edge - NIR)}{(Red\ Edge + NIR)}$	H. Wu et al. (2019)
**NDRE**	Normalized Difference Red Edge Index	$\frac{(NIR - Red\ Edge)}{(NIR + \ Red\ Edge)}$	Barnes et al. (2000)
**NDVI**	Normalized Difference Vegetation Index	$\frac{(NIR - Red)}{(NIR + \ Red)}$	Rouse Jr et al. (1974)
**NDVIG**	Normalized Difference Vegetation Index - Green	$\frac{(NIR - Green)}{(NIR + \ Green)}$	Jiang et al. (2013)
**NDWI**	Normalized Difference Water Index	$\frac{(Green - NIR)}{(Green + NIR)}$	Gao (1996)
**NGRDI**	Normalized Difference Green/Red Index	$\frac{(Green - Red)}{(Green + Red)}$	Gitelson et al. (2002)
**RI**	Redness Index	$\frac{(Red - Green)}{(Red + Green)}$	Escadafal and Huete (1991)
**RVI**	Ratio Vegetation Index	$\frac{R}{NIR}$	Pearson and Miller (1972)
**SR**	Simple Ratio	$\frac{NIR}{Red}$	Jordan (1969)

**VARI**	Visual Atmospheric Resistance Index	$\frac{(Green - Red)}{(Green + Red - Blue)}$	Gitelson et al. (2002)

Table S3. Average and median value of model evaluation metrics (mean absolute error (MAE), root mean squared error (RMSE) and r-squared) from 100 independent models of digestible nitrogen

Metric	Test	Mean	Median	Standard deviation	Standard error
**MAE**	Cross-validation	0.103	0.103	0.0025	0.0002
**MAE**	Independent validation	0.168	0.166	0.0175	0.0018
**RMSE**	Cross-validation	0.103	0.148	0.0090	0.0009
**RMSE**	Independent validation	0.223	0.223	0.0283	0.0028
**R^2**	Cross-validation	0.103	0.610	0.0752	0.0075
**R^2**	Independent validation	0.036	0.019	0.0429	0.0043

Table S4. Number of leaf samples per species.

Species	Number of samples
Eucalyptus botryoides	1
Eucalyptus consideniana	10
Eucalyptus croajingolensis	15
Eucalyptus cypellocarpa	20
Eucalyptus delegatensis	4
Eucalyptus elata	2
Eucalyptus fastigata	8
Eucalyptus globoidea	11
Eucalyptus mackintii	1
Eucalyptus muelleriana	6
Eucalyptus nitens	6
Eucalyptus obliqua	31
Eucalyptus polyanthemos	4
Eucalyptus pseudoglobulus	6
Eucalyptus sieberi	14
Eucalyptus tricarpa	2
Eucalyptus viminalis	11

References

Barnes, E., Clarke, T., Richards, S., Colaizzi, P., Haberland, J., Kostrzewski, M., . . . Thompson, T. (2000). *Coincident detection of crop water stress, nitrogen status and canopy density using ground based multispectral data.* Paper presented at the Proceedings of the Fifth International Conference on Precision Agriculture, Bloomington, MN, USA.

Escadafal, R., & Huete, A. (1991). Etude des propriétés spectrales des sols arides appliquée à l'amélioration des indices de végétation obtenus par télédétection. *Comptes rendus de l'Académie des sciences. Série 2, Mécanique, Physique, Chimie, Sciences de l'univers, Sciences de la Terre, 312*(11), 1385-1391.

Gao, B.-c. (1996). NDWI—A normalized difference water index for remote sensing of vegetation liquid water from space. *Remote Sensing of Environment, 58*(3), 257-266. doi:https://doi.org/10.1016/S0034-4257(96)00067-3

Gitelson, A. A., Kaufman, Y. J., Stark, R., & Rundquist, D. (2002). Novel algorithms for remote estimation of vegetation fraction. *Remote Sensing of Environment, 80*(1), 76-87.

Jiang, N., Zhu, W., Zheng, Z., Chen, G., & Fan, D. (2013). A comparative analysis between GIMSS NDVIg and NDVI3g for monitoring vegetation activity change in the northern hemisphere during 1982–2008. *Remote Sensing, 5*(8), 4031-4044.

Jordan, C. F. (1969). Derivation of leaf-area index from quality of light on the forest floor. *Ecology, 50*(4), 663-666.

Motohka, T., Nasahara, K. N., Oguma, H., & Tsuchida, S. (2010). Applicability of green-red vegetation index for remote sensing of vegetation phenology. *Remote Sensing, 2*(10), 2369-2387.

Nedkov, R. (2017). Normalized Differential Greenness Index for vegetation dynamics assessment. *Comptes rendus de l'Academie bulgare des Sciences, 70*(8), 1143-1146.

Pearson, R. L., & Miller, L. D. (1972). Remote mapping of standing crop biomass for estimation of the productivity of the shortgrass prairie. *rse*, 1355.

Rouse Jr, J., Haas, R., Schell, J., & Deering, D. (1974). Monitoring vegetation systems in the Great Plains with ERTS.

Wu, H., Levin, N., Seabrook, L., Moore, B. D., & McAlpine, C. (2019). Mapping Foliar Nutrition Using WorldView-3 and WorldView-2 to Assess Koala Habitat Suitability. *Remote Sensing, 11*(3). doi:10.3390/rs11030215

Wu, W. (2014). The generalized difference vegetation index (GDVI) for dryland characterization. *Remote Sensing, 6*(2), 1211-1233.
